# The wrong horse was bet on: the effects of argument structure versus argument adjacency on the processing of idiomatic sentences

**DOI:** 10.3389/fpsyg.2023.1123917

**Published:** 2023-05-04

**Authors:** Laura Reimer, Eva Smolka

**Affiliations:** ^1^Institute of German Studies, University of Münster, Münster, Germany; ^2^Department of Psychology, University of Konstanz, Konstanz, Germany; ^3^Department of Linguistics, University of Vienna, Vienna, Austria

**Keywords:** idiomatic sentences, figurative meaning, syntactic processing, passive voice, argument structure, number of arguments, adjacency

## Abstract

**Introduction:**

Psycholinguistic research remains puzzled about the circumstances under which syntactically transformed idioms keep their figurative meaning. There is an abundance of linguistic and psycholinguistic studies that have examined which factors may determine why some idioms are more syntactically fixed than others, including transparency, compositionality, and syntactic frozenness; however, they have returned inconclusive, sometimes even conflicting, results. This is the first study to examine argument structure (i.e., the number of arguments a verb takes) and argument adjacency (i.e., the position of the critical arguments relative to the verb) and their effects on the processing of idiomatic and literal sentences in German. Our results suggest that neither the traditional models of idiom processing (according to which idioms are stored as fixed entries) nor more recent hybrid theories (which concede some compositional handling in addition to a fixed entry) adequately account for the effects of argument structure or argument adjacency. Therefore, this study challenges existing models of idiom processing.

**Methods:**

In two sentence-completion experiments, participants listened to idiomatic and literal sentences in both active and passive voice without the sentence-final verb. They indicated which of three visually-presented verbs best completed the sentence. We manipulated the factor argument structure within experiments and argument adjacency across experiments. In Experiment 1, passivized three-argument sentences had the critical argument adjacent to the verb while two-argument sentences had the critical argument non-adjacent to the verb, and vice versa in Experiment 2.

**Results:**

In both experiments, voice interacted with argument structure. Active sentences—both literal and idiomatic—showed equivalent processing of two- and three-argument sentences. However, passive sentences returned contrasting effects. In Experiment 1, three-argument sentences were processed faster than two-argument sentences and vice versa in Experiment 2. This pattern corresponds to faster processing when critical arguments are adjacent than non-adjacent.

**Discussion:**

The results point to the dominant role of argument adjacency over the number of arguments in the processing of syntactically transformed sentences. Regarding idiom processing, we conclude that the adjacency of the verb to its critical arguments determines whether passivized idioms keep their figurative meaning and present the implications of this finding for relevant models of idiom processing.

## 1. Introduction

During sentence processing, different types of information are used to construct the meaning of the sentence as a whole. The verb is a valuable source of information in this process as it is at the interface between semantics and syntax and carries more information than other words in a sentence. One such type of information is the *lexical representation* of the verb (e.g., Healy and Miller, 1970; Levelt, 1989; Pickering and Branigan, 1998) which, it is assumed, includes the argument structure of the verb, that is, the number of arguments a verb takes. The verb *to give*, for instance, requires three arguments, namely a subject, a direct object, and an indirect object, as in “*Pat gave a cookie to Kim*” (e.g., Bencini and Goldberg, 2000). Verbs such as *to give*, which require three arguments, are called ditransitive verbs, while verbs that require two arguments, such as *to eat*, as in “*Pat ate a cookie*”, are called transitive verbs. Finally, verbs that require one argument, such as *to run*, as in “*Pat ran*”, are referred to as intransitive verbs. However, as verbs occur in many argument structure configurations (e.g., Goldberg, 1995), it is difficult to construct the meaning of a sentence based solely on information contained within the verb. The verb *kick*, for instance, which is considered a prototypical transitive verb, can occur in at least eight argument structure frames (Bencini and Goldberg, 2000). Examples are “*Pat kicked the wall*”, which includes a transitive action, “*Pat kicked the football into the stadium*”, which includes a caused motion, and “*Horses kick*”, which includes an intransitive action. Therefore, Bencini and Goldberg (2000) have shown that people do not rely solely on verb information to interpret the meaning of a sentence, rather the configuration of the verb’s arguments also plays a crucial role in the interpretation of the sentence.

Idiomatic sentences are considered special when it comes to the reliability of verb information and the configuration of verb arguments. First, the meaning of the verb in an idiomatic sentence often deviates far from its prototypical meaning. For instance, in addition to occurring in different argument structure frames, the meaning of the verb “*kick*” has a completely different meaning when it is part of the idiomatic sentence “*Pat kicked the bucket*”. This sentence does not have the same prototypical transitive action as “*Pat kicked the wall*”: it has the intransitive action of *to die*. Second, the argument structure of the verb in an idiomatic sentence often deviates from the prototypical argument structure of the same verb in a literal sentence. For instance, the German verb “*öffnen*” (literal (L): *to open*) is a transitive verb that requires both a subject and an object. However, in the German idiom “*jemandem die Augen öffnen*” (L: *to open somebody’s eyes*; figurative (F): “*to tell someone a few home truths*”), the idiomatic reading requires an additional indirect object (here: *somebody*). If this indirect object is not present, the figurative meaning is lost. That is, the number of arguments a verb takes is critical to idiom processing. This is the first study that considers the number of arguments in an idiom, their configuration, and, specifically, whether or not the arguments are adjacent to the verb as potential factors influencing idiom processing.

Idioms exhibit a special semantic and syntactic behavior, as demonstrated below, and this study focuses on their syntactic processing. Traditionally, idiomatic sentences such as “*to kick the bucket*” or “*to reach for the stars*” are assumed to be semantically fixed, meaning that their individual parts cannot be substituted without the figurative meaning of the idiom being lost, as in “*he kicked the pail*” or “*she reached for the planets*” (e.g., Moon, 1998). In addition to their semantic fixedness, idioms are also considered syntactically fixed because they cannot undergo the full range of syntactic transformations that can be applied to literal sentences, as in “*the stars were reached for by her*”, “*it was the stars she reached for*”, or “*she reached for the bright stars*” (e.g., Jackendoff, 1997). Such semantic and syntactic peculiarities need to be accounted for. Indeed, numerous linguistic and psycholinguistic studies have examined factors, including transparency, compositionality, and syntactic frozenness, that may determine why some idioms are more syntactically fixed than others. For example, an idiom is considered transparent when there is a rationale for the figuration, as in “*to saw logs*” meaning “*to snore*” (e.g., Gibbs and Nayak, 1989; Nunberg et al., 1994). Regarding compositionality, the idiom “*spill the beans*” with the meaning “*to divulge the information*” is considered decomposable as “*spill*” denotes the act of divulging and “*beans*” the information that is being divulged (Nunberg et al., 1994; see also Gibbs and Nayak, 1989; Cutting and Bock, 1997; Peterson et al., 2001; Sprenger et al., 2006; Titone and Libben, 2014; Kyriacou et al., 2020). Finally, on syntactic frozenness, frozen idioms cannot undergo even the simplest transformations without losing their figurative interpretation, as is the case for “*Some steam was blown off at the party*” (Fraser, 1970; see also Gibbs and Gonzales, 1985; Peterson et al., 2001). However, these previous studies have returned inconclusive results. For example, while Gibbs and Nayak (1989) observed that decomposable idioms are syntactically more flexible than non-decomposable idioms, other studies did not confirm these results and some have even found the opposite (see Cutting and Bock, 1997; Peterson et al., 2001; Sprenger et al., 2006; Titone and Libben, 2014; Kyriacou et al., 2020). Thus, Gibbs and Gonzales (1985) found that syntactically frozen idioms cannot be transformed at all, while Peterson et al. (2001) concluded that all idioms—be they syntactically flexible or frozen—are parsed by the syntactic processor. Furthermore, Titone and Libben (2014) have suggested that multiple linguistic factors jointly constrain figurative meaning retrieval in a time-dependent fashion. This demonstrates, as stated above, that the current findings are, at best, inconclusive. To the best of our knowledge, this is the first study to examine argument structure and argument adjacency as potential factors that influence the syntactic behavior of idioms. In the following, we review some of the most influential models of idiom processing with a particular focus on their potential to predict the influence of argument structure and argument adjacency. We demonstrate that neither of these currently dominant models account for the influences of argument structure and argument adjacency and explain how the present study challenges these models.

### 1.1. Models of idiom processing

Traditional theoretical accounts capture the fixedness of idioms as representations in the mental lexicon, where idioms are stored as whole phrases and each phrase has its own semantic entry (e.g., Chomsky, 1980; Levelt, 1989; Moon, 1998; Jackendoff, 2008). This assumption is reflected in the form of non-compositional processing in traditional psycholinguistic models, such as the *idiom list* hypothesis (Bobrow and Bell, 1973), the *lexical representation* hypothesis (Swinney and Cutler, 1979), and the *direct access* hypothesis (Gibbs, 1980). Due to the fixedness of the entry, neither argument structure nor argument adjacency are expected to play a role in these processing accounts.

However, newer hybrid models incorporated the assumption that the compositional handling of an idiom affects its comprehension just as its non-compositional representation does (see Cacciari and Tabossi, 1988; Cutting and Bock, 1997; Sprenger et al., 2006; Libben and Titone, 2008; Tabossi et al., 2009; Titone and Libben, 2014; Cacciari and Corradini, 2015). The compositional process is reflected in the assumption that the figurative meaning is determined based on the literal meaning of the individual constituents of the idiomatic phrase. Thus, according to the configuration hypothesis (Cacciari and Tabossi, 1988), the interpretation of a sentence is completely literal up to a point referred to as the idiom “key”. At this point, the figurative meaning is recognized and understood from that point on. As the figurative meaning is related to the recognition of a specific configuration, when the idiom key occurs earlier in the sentence, this should result in earlier recognition of the idiom compared to sentences in which the idiom key occurs later. Hence, argument structure (the number of arguments) should play a significant role in idiom processing, though argument adjacency may not. Consider the following iterations of the German idiom “*nach den Sternen greifen*” (L: *to reach for the stars*; F: “*to try to achieve something impossible*”) as written in the perfect tense, which requires an auxiliary verb in the second position and the lexical verb (the participle) is given in sentence-final position: “*Sie hat nach den Sternen gegriffen*” (L: *She reached for the stars*) versus “*Nach den Sternen hat sie gegriffen*” (L: *For the stars she reached*). In the former, the critical idiomatic argument “*nach den Sternen*” is adjacent to the verb, while in the latter it is non-adjacent to the verb but occurs early in the sentence. According to the configuration hypothesis, the figurative meaning should be recognized faster in the latter example, because the critical argument, which functions as the idiom key, occurs early in the sentence.

The production model by Cutting and Bock (1997) is another important hybrid model that attempts to integrate the syntactic properties of the otherwise compositional idioms in form of phrasal frames. This model is theoretically extended by the superlemma theory (Sprenger et al., 2006), which integrates a fixed representation of the syntactic properties of the idiom in the form of a “superlemma.” According to the superlemma theory, the information relevant to the syntactic use of an idiom is idiosyncratic and must, therefore, be encoded in the lexicon. As a consequence of its idiosyncratic nature, idiom syntax must be learned through experience. Thus, the superlemma model imposes syntactic restrictions on idiomatic structures and incorporates the syntactic peculiarities related to specific idiomatic phrases. However, it does not explain why some idioms are more syntactically flexible than others.

In an eye-tracking study, Holsinger (2013) observed that idiomatic processing is not restricted to sentence boundaries, as it should be according to the notion of a superlemma. Instead, they found that their participants activated the figurative meaning even when the syntactic environment was incompatible with the idiom, such as when the critical words (e.g., *kick* and *bucket*) occur across sentence boundaries (e.g., … *missed the ball when he kicked. The bucket full of orange slices was* …). Therefore, these findings question the role of the superlemma, which should restrict the figurative reading to a specific syntactic structure. Furthermore, and in contrast to the predictions of the hybrid models, more recent evidence has shown that the activation of the literal meaning is not terminated as soon as the figurative meaning is recognized, it actually remains active during the processing of the entire sentence (Smolka et al., 2007a; Rabanus et al., 2008). These findings are the main feature of purely compositional accounts such as the stem-based frequency account (e.g., Smolka et al., 2007b; Rabanus et al., 2008). Furthermore, more recent studies on the processing of idiomatic sentences have demonstrated that idioms are not as semantically fixed as was previously assumed (e.g., Holsinger, 2013; Geeraert et al., 2017; Smolka and Eulitz, 2020). For example, the stem-based frequency model (Smolka et al., 2007a, 2014; Rabanus et al., 2008; Smolka and Eulitz, 2020) explains why the individual constituents of certain idioms (e.g., “*stars*” in “*to reach for the stars*”) can be substituted with semantically associated words (e.g., “*planets*”) without losing their figurative meaning. This model assumes the same representations and processes for both idiomatic and literal configurations (see also Kyriacou et al., 2020; Mancuso et al., 2020). Thus, individual stems (i.e., word constituents) are accessed and their associated mental concepts are activated. The frequency of a particular constituent determines the strength of meaning activation at the concept level and particular constituent combinations activate their joint concept (also idiomatic combinations). For instance, the combination “*reach*” and “*stars*” will activate the concept that expresses the figurative meaning “*to try to achieve something impossible*”. The meaning of this concept will further activate the closely related concept, ambition. The frequency of a particular constituent combination (e.g., “*reach*”, “*for*”, and “*stars*”) determines how strongly the common concept will be activated. Furthermore, as the constituents will always activate their closely related concepts, the literal-meaning associations will be available regardless of the meaning of the entire phrase, which may explain the lexical flexibility of idioms. As the figurative concept is activated by co-occurring constituents, argument adjacency should play a vital role in idiom recognition according to this account. Argument structure, however, should only affect idiom processing if the critical arguments are adjacent.

In this study, we have focused on the syntactic processing of idiomatic sentences and, in particular, on factors that influence the syntactic fixedness of idioms. To this end, we conducted two sentence completion experiments to investigate how verb information (i.e., argument structure) and the adjacency of the critical arguments relative to the verb influences the processing of idiomatic sentences. To our knowledge, this is the first study that directly compares the effects of argument structure and argument adjacency on the processing of idiomatic sentences, in active or passive voice. In addition, we examined the effects of argument structure on literal sentences in German, also in both active and passive voice.

## 2. Experiment 1

In Experiment 1, we investigated the influence of verb information and compared the processing of verbs with different numbers of arguments: transitive verbs with two arguments (subject and direct object) and ditransitive verbs with three arguments (subject, direct object, and indirect object). Previous research on the effects of the number of arguments has been conducted on literal language and studied language acquisition and language production in agrammatic patients and unimpaired participants. The findings have shown, that, in general, the number of arguments required by a verb affects sentence production in that the higher number of arguments there are in a sentence, the higher the number of errors (Thompson et al., 1995, 1997; De Bleser and Kauschke, 2003). Thus, sentences with one argument (e.g., ‘*[he] sits*’)^1^ are easier to produce than sentences with two arguments (e.g., “*[he] fixes [the chair]*”), which are again easier to produce than sentences with three arguments (e.g., “*[he] gives [her] [the present]*”). According to Shapiro et al. (1987, 1989), the complexity of the argument structure also affects language comprehension. In their study, lexical decision times in healthy participants were slower for verbs like “*to give*”, which requires three arguments, than for verbs like “*to fix*”, which requires only two arguments. In the present study, we examined whether the effect of argument structure also holds for literal sentence processing in German.

While verb properties have been shown to play an important role in the study of literal language, they have, to date, been neglected in the psycholinguistic study of figurative language. Indeed, most studies on idiom processing (e.g., Gibbs and Nayak, 1989; Peterson et al., 2001; Tabossi et al., 2009) have not manipulated argument structure and have used a mixture of verbs that require two and three arguments (e.g., “*[he] hits [the spot]*”, “*[he] plays [second fiddle] [to someone]*”). Few studies (e.g., Gibbs et al., 1989; Holsinger, 2013) have controlled for the number of arguments and have employed idioms with only two arguments. To our knowledge, this is the first study that has directly manipulated the verb argument structure of idiomatic sentences and compared the processing of idiomatic sentences containing two arguments with those containing three arguments. There are several differences between idioms containing two and three arguments. First, in most idiomatic sentences with three arguments of the type “*[subject] verb [indirect object] [direct object]*”, as in “*[Sie] hat [der Freundin] [die Augen] geöffnet*” (word-by-word (W): “*[she] has [the friend’] [the eyes] opened*”; L: *She opened the friend’s eyes*; F: “*She told the friend a few home truths*”), the content of the indirect object (here, *der Freundin, the friend_*DAT*_*) does not contribute to the figurative meaning, that is, it does not directly serve as an idiomatic constituent because this position can be arbitrarily filled (e.g., *the sister_*DAT*_, the teacher_*DAT*_*). Nevertheless, the figurative reading requires this indirect object because its omission leads, in most cases, to a literal non-figurative reading (“*[Sie] hat [die Augen] geöffnet*”, L: *She opened the eyes*) that corresponds to the transitive form of the verb “*öffnen*” (L: *to open*). Thus, although a semantic analysis of the indirect object is not necessary for this idiom, the syntactic system must include this argument to enable the figurative reading and this, in turn, causes a so-called “syntactic-semantic mismatch” in idioms that contain three arguments (see Peterson et al., 2001 for syntactic-semantic mismatch).

Second, an idiomatic sentence with two arguments, such as “*[Sie] hat [nach den Sternen] gegriffen*” (L: *She reached for the stars*), usually features a literal subject (“*Sie*”) and an idiomatic direct object (“*nach den Sternen*”), whereas an idiom with three arguments, such as “*[Sie] hat [der Freundin] [die Augen] geöffnet*”, often has a literal subject (“*Sie*”), a literal indirect object (“*der Freundin*”), and an idiomatic direct object (“*die Augen*”). That is, idioms with two arguments have only one literal argument, while idioms with three arguments often have two literal arguments, meaning that idioms with three arguments often contain more literal arguments than idioms with two arguments. Third, German sentences with three arguments usually have a typical order: subject (e.g., “*Sie*”, *she*), indirect object (e.g., “*der Freundin*”, *the friend’s*), direct object (e.g., “*die Augen*”, “*the eyes*”). Hence, in idiomatic sentences with three arguments, the idiomatic key—the critical argument for recognition of the figurative meaning (e.g., Cacciari and Tabossi, 1988)—occurs rather late in the sentence. For example, after hearing the words “*Sie hat der Freundin*…”, word-for-word: “*she has the friend’s*…”, there is still no hint that the sentence will become idiomatic. Thus, it is expected that the activation of the figurative meaning occurs later in sentence processing and it is possible that the literal analysis is more prominent in idiomatic sentences with three arguments than in idiomatic sentences with two arguments. Furthermore, the prototypical passive sentence features a verb that requires two arguments (see Eisenberg, 2006:125), suggesting that this type of sentence might have a processing advantage over other passive sentences.

To summarize, there are several reasons why idiomatic sentences with two or three arguments may be processed differently, especially when they are passivized. In order to examine this, we constructed pairs of sentences containing the same verb (see Table 1): one with a figurative reading, such as “*Sie hat dem Jungen den Kopf gewaschen*” (L: *She washed the boy’s head; F*: “*She gave the boy a piece of her mind*”), the other with a literal reading, such as “*Er hat der Tochter die Haare gewaschen*” (L: *He washed the daughter’s hair*). In a speeded sentence completion task, participants listened to sentences without the sentence-final verb (i.e., up to the word before the last word of the sentence) and then chose which of three visually presented verbs best completed the sentence. One verb was a completion that triggered the figurative meaning, which we will refer to below as the “figurative verb”, (e.g., “*gewaschen*”; L: *washed*), the second verb was a semantic associate of the figurative verb (e.g., “*gereinigt*”; L: *cleaned*), and the third was an unrelated literal control (e.g., “*gemessen*”; L: *measured*). Most importantly, all three verbs represented meaningful readings of the sentence. For literal sentences, all verb types (i.e., figurative, semantic associate, unrelated) were semantically plausible ways to complete the sentence. The group of three choices provided for each sentence are referred to below as the “verb triplet”. As Table 1 demonstrates, the idiomatic and literal sentence pairs had an argument structure with either two or three arguments and the sentences were presented in either active or passive voice.

**TABLE 1:** Examples of idiomatic and literal sentence pairs holding the same verb, with two and three arguments, presented in active and passive voice, and the corresponding verb targets.

Idiomatic	Literal	Figurative	Associate	Unrelated
		Target verbs		
**Sentence pairs with two arguments**				
**Active**				
*Sie hat immer nach den Sternen gegriffen.* (W) She has always for the stars reached. (L) She always reached for the stars. (F) She tried to achieve something impossible.	*Das Mädchen hat nach den Bonbons gegriffen.* (W) The girl has for the sweets reached. (L) The girl reached for the sweets.	*gegriffen* (“reached”)	*gelangt* (“grabbed”)	*gelebt/getastet* (“lived”/“fumbled”)
**Passive**				
*Nach den Sternen wurde immer von ihr gegriffen.*	*Nach den Bonbons wurde von dem Mädchen gegriffen.*			
**Sentence pairs with three arguments**				
**Active**				
*Sie hat dem Jungen den Kopf gewaschen.* (W) She has the boy the head washed. (L) She washed the boy’s head. (F) She gave the boy a piece of her mind.	*Er hat der Tochter die Haare gewaschen.* (W) He has the daughter the hair washed. (L) He washed the daughter’s hair.	*gewaschen* (“washed”)	*gereinigt* (“cleaned”)	*gemessen/gebürstet* (“measured”/“brushed”)
**Passive**				
*Dem Jungen wurde von ihr der Kopf gewaschen.*	*Der Tochter wurden von ihm die Haare gewaschen.*			

Translations of a sentence into English are given: word-by-word (W), literal (L), and figurative (F).

Our investigation focused on two questions that have been central in the psycholinguistic literature on sentence processing in general and idiom processing in particular. *Question 1: Does sentence processing, in particular that of idiomatic sentences, differ depending on whether they are in active or passive voice?* Seminal studies on literal sentence processing have demonstrated that there is a general preference for active over passive sentences (Mehler, 1963) and that passive sentences are more difficult to understand than active sentences (Ferreira, 2003). Furthermore, there is a processing advantage for active over passive sentences when certain conditions hold, such as when the event is coded in terms of the actor or when the hearer’s attention is directed to the actor as the logical subject (Tannenbaum and Williams, 1968; Olson and Filby, 1972). However, a more recent study using self-paced reading found that passive sentences were processed faster than active sentences, although the passive structure did induce more comprehension errors, which suggests there may be a speed-accuracy trade-off (Paolazzi et al., 2019). In relation to specifically idiomatic sentence processing, we are aware of only one other study that has compared the processing of active and passive sentences. Kyriacou et al. (2020) used eye-tracking to confirm previous findings that active idiomatic sentences are processed faster than passive idiomatic sentences. Based on these studies, we expected to find a processing advantage of active over passive sentences in general and, if this processing advantage generalizes to idiomatic sentences, we expected to also identify a processing advantage for active idiomatic sentences over passive idiomatic sentences. *Question 2: Does the number of arguments a verb takes affect sentence processing in general and that of idiomatic sentences in particular?* It is generally assumed that literal sentences with two arguments are easier to process than literal sentences with three arguments, as their argument structure is less complex (e.g., Thompson et al., 1995, 1997). If this effect of argument structure in literal sentences generalizes to idiomatic sentences, idiomatic sentences with two arguments will be processed faster than idiomatic sentences with three arguments. Such a finding would also support the assumption that idiomatic sentences are fully parsed and syntactically analyzed through the same process that literal sentences are (see Peterson et al., 2001; Tabossi et al., 2009). We further hypothesized that if the number of arguments a verb takes influences the syntactic fixedness of idiomatic sentences, the passivization of sentences with three arguments will be less disruptive to the figurative meaning than of sentences with two arguments because the former are more “literal” and contain two literal objects, while the latter contain only one literal object. If, however, the number of arguments does not influence the syntactic fixedness of idioms, passivization will lead to a loss of figurative meaning in idiomatic sentences with two and three arguments alike.

To summarize, by manipulating the Sentence Type (idiomatic/literal) we compared the underlying processes used to interpret idiomatic and literal sentences. Manipulating the voice (active/passive) enabled us to examine whether idiomatic sentences can be passivized and still retain their figurative meaning. Manipulating the number of arguments (two/three) allowed us to examine whether the argument structure effect on literal sentences generalizes to idiomatic sentences and, finally, whether the number of arguments affects the passivization of idiomatic sentences.

### 2.1. Materials and methods

#### 2.1.1. Participants

Thirty-one students from the University of Konstanz (12 male, 19 female, mean age of 25.5 years) were paid five euros to participate in the experiment. All were native German speakers. No participants were dyslexic and all had normal or corrected-to-normal vision.

#### 2.1.2. Materials

Supplementary appendix A lists all the stimuli used in Experiment 1. To ensure that all idiomatic expressions in passive voice were recognizable as idioms, they were selected from the sentence pool that was tested in the sentence completion task described below.

#### 2.1.3. Pretest 1: sentence completion task on idioms in the passive voice

We selected 197 German idioms in the active voice from the *Dictionary of German Idiomaticity* (Redewendungen, 2008). Of these, 75 contained two arguments and 122 contained three arguments. In the sentences with two arguments, the idiomatic expressions featured one object (e.g., “*[nach den Sternen] greifen*”; L: *to reach [for the stars]*; F: “*to try to achieve something impossible*”); in the sentences with three arguments, the idiomatic expressions contained two objects (e.g., “*[jemandem] [den Kopf] waschen*”; L: *to wash [someone’s] [head]*; F: “*to give someone a piece of one’s mind*”), only one of which was critical to the idiomatic meaning. Each idiomatic expression in the active voice was then transformed into the canonical passive voice (see Burchert and de Bleser, 2010 for information on the canonical passive voice). Table 1 presents the examples and their translations. For instance, the two-argument sentence “*Sie hat nach den Sternen gegriffen*” became “*Nach den Sternen wurde von ihr gegriffen*” and the three-argument sentence “*Sie hat dem Jungen den Kopf gewaschen*” became “*Dem Jungen wurde von ihr der Kopf gewaschen*”. The 197 passivized sentences were distributed over six questionnaires, four of which contained only three-argument sentences and two of which contained only two-argument sentences. The same number of literal sentences in the passive and containing the same number of arguments were added to the questionnaires to yield a proportion of 50% idiomatic and 50% literal sentences. For the sentence completion task, we followed the same procedure as Smolka et al. (2007a) and cast all sentences in the perfect tense. We presented participants with the main verb’s participle prefix, *ge*-, for example, “*Dem Jungen wurde von ihr der Kopf ge*_____”, in order to exclude completions with complex verbs (i.e., prefix and particle verbs, which are very common in German). The participant’s task was to complete each item with the verb that best fits the sentence. In total, 120 German native speakers participated in this pre-test. The questionnaires were distributed either in electronic form (via email) or printed form. We then counted the number of idiomatic completions for each sentence, that is, how often participants specified the verb that provided the figurative meaning of the sentence.

#### 2.1.4. Experimental sentences

From this sentence pool, we selected 52 idiomatic sentences that fulfilled the following criteria: (a) the passivized idiom had a sentence completion rate higher than 20% (with a range between 20 and 100%) so that the sample included both more and less syntactically frozen idioms, (b) the idiom could be completed by verbs that force a literal meaning on the sentence, and (c) the verb that completed the idiomatic sentence occurred only once in our experimental set. All idiomatic sentences were ambiguous (i.e., they had both a plausible literal and a figurative meaning). Each of the 52 idiomatic sentences were paired with a literal sentence that held the same main verb, resulting in 52 sentence pairs, for example, figurative (F) “*Sie hat nach den Sternen gegriffen*” (L: “*She reached for the stars*”) and literal (L) “*Das Mädchen hat nach den Bonbons gegriffen*” (L: *The girl reached for the sweets*). Of the 52 sentence pairs, 24 contained two arguments (“*[Sie] hat [nach den Sternen] gegriffen*”) and 28 contained three arguments (“*[Sie] hat [dem Jungen] [den Kopf] gewaschen*”). All sentences were presented in both active and passive voice. All passivized sentences followed the canonical sentence structure (i.e., order of arguments). Sentences with two arguments, by definition, have one less argument than sentences with three arguments and, therefore, our controlled examples consisted of fewer words. To ensure the same number of words across all sentences in active voice, adjectives and adverbs were added to the sentences with two arguments (“*Sie hat nach den Sternen gegriffen*” became “*Sie hat immer nach den Sternen gegriffen*”, L: *She always reached for the stars*). All sentences in the active voice contained seven words and all sentences in the passive voice contained eight words. All sentences were presented in the perfect tense with the auxiliary verb in the second position and the main verb in the sentence-final position. Each pair of sentences (a) were completed by the same verb, for example, both the idiomatic sentence “*Sie hat dem Jungen den Kopf gewaschen*” and the literal sentence “*Er hat der Tochter die Haare gewaschen*” end with *gewaschen* and, thus, (b) had the same argument structure. Each sentence was presented in active and in passive voice, resulting in 52 sentence quartets (see Table 1).

#### 2.1.5. Pretest 2: sentence completion task on idioms in active voice

To ensure that all idiomatic and literal sentences were equally comprehensible, we collected sentence completion data for the final set of 52 idiomatic and 52 corresponding literal sentences in active voice. Again, the main verb’s participle prefix, *ge*-, was provided and participants were asked to complete each item with the verb that best fitted the sentence. Sentences were randomly distributed over the questionnaire. Twenty-two German native speakers who had not taken part in Pretest 1 participated in Pretest 2. The verb that rendered the figurative meaning, henceforth “figurative verb,” was used to complete 76% of the idiomatic sentences: 82% of the sentences with two arguments and 72% of the sentences with three arguments. Furthermore, the literal sentences had a high completion rate with the “figurative verb.” The overall completion rate was 52%, 54% for sentences with two arguments and 50% for sentences with three arguments. These figures confirm that the sentence-final verbs were highly predictable, more so than in previous studies on idiom processing. For example, in their seminal experiments, Cacciari and Tabossi (1988) applied altogether nine ambiguous idioms with completion rates of 45% (as compared with 76% in our study).

#### 2.1.6. Targets

A verb triplet containing three types of verb targets were selected as possible completions for each idiomatic sentence: (a) the verb associated with the figurative meaning, hereafter the “figurative verb,” for example, “*gegriffen*” (L: *reached*) for the sentence “*Sie hat immer nach den Sternen*____,” (b) a semantic associate of the figurative verb, in this example “*gelangt*” (L: *grabbed*), and (c) a verb unrelated to the figurative verb, in this example “*gelebt*” (L: *lived*). All verbs completed the sentence in a meaningful way: (a) generated a figurative reading, while (b) and (c) generated literal readings. The paired literal sentences could be completed by options given in a second verb triplet that contained (a) the same figurative verb, such as “*gegriffen*” (L: *reached*) in the sentence “*Das Mädchen hat nach den Bonbons*____,” (b) the same semantic associate of the figurative verb “*gelangt*” (L: *grabbed*) and (c) an unrelated verb that was sometimes different from the option provided in the verb triplet for the idiomatic sentence, for example, “*getastet*” (L: *fumbled*) differed from the third option presented in the idiomatic sentence’s verb triplet. All three verbs in the verb triplets for the literal sentences resulted in the sentence having a plausible and correct literal meaning. The three verbs included in each verb triplet were as closely matched as possible in terms of the number of letters, number of syllables, and their lemma frequency (according to CELEX, Baayen et al., 1993). See Table 2 for a complete list of matched variables for the verb triplets.

**TABLE 2:** Stimulus characteristics of the verb triplets that complete the idiomatic and literal sentences.

	Idiomatic			Literal		
	Figurative	Associate	Unrelated	Figurative	Associate	Unrelated
Example	*gegriffen*	*gelangt*	*gelebt*	*gegriffen*	*gelangt*	*getastet*
**Two arguments**						
Lemma Abs	1424 (2,109)	635 (1,588)	1,510 (2,460)	1,424 (2,109)	784 (1,702)	1,549 (2,431)
Lemma ML	239 (354)	107 (267)	254 (413)	239 (354)	132 (286)	260 (408)
Syllables	1.7 (0.5)	1.3 (0.4)	1.5 (0.5)	1.7 (0.5)	1.6 (0.5)	1.6 (0.5)
Letters	8.3 (1.1)	7.6 (1.2)	8.3 (1.4)	8.3 (1.1)	8.3 (1.3)	7.9 (0.9)
**Example**	***gewaschen***	***gereinigt***	***gemessen***	***gewaschen***	***gereinigt***	***gebürstet***
**Three arguments**						
Lemma Abs	792 (898)	994 (2,219)	478 (552)	793 (898)	943 (2,413)	293 (384)
Lemma ML	189 (395)	167 (373)	80 (93)	189 (395)	159 (405)	49 (64)
Syllables	1.6 (0.5)	1.5 (0.5)	1.3 (0.5)	1.6 (0.5)	1.5 (0.5)	1.5 (0.5)
Letters	8.2 (1.2)	8.2 (1.4)	7.7 (1.1)	8.2 (1.2)	8.3 (1.3)	7.9 (1.1)

Lemma Abs, absolute mean lemma frequency; Lemma ML, mean lemma frequency per one million; both taken from CELEX (Baayen et al., 1993); Syllables, mean number of syllables; Letters, mean number of letters; SD in parentheses.

#### 2.1.7. Filler sentences and targets

We added 156 literal sentence pairs as fillers; of these, 72 sentence pairs contained two arguments, and 84 contained three arguments. All literal filler sentences were cast in both active and passive voice. All sentences featured verbs other than the verbs of the experimental set. The active and passive versions of the same sentence pair were allocated to two lists by means of a Latin square design that yielded 156 literal filler sentences for each list. The high proportion of filler sentences resulted in a ratio of idiomatic to literal sentences of 20:80 and a ratio of experimental to filler sentences of 40:60.

#### 2.1.8. Apparatus

The complete sentences were recorded in a quiet and neutral manner by a female German native speaker, a 36-year-old woman from Frankfurt. The recording took place in a sound-attenuated cabin in the Phonological Lab at the University of Konstanz. The length of the audio files was standardized using the PRAAT software package (Boersma and Weenink, 2009). The auditory stimuli were presented to participants via Sennheiser headphones (HD 595) while the visual stimuli were presented on a 17′ flat-screen monitor (Flatron L1810B) connected to a BEST personal computer. Response latencies were recorded with a key press on a three-button box connected to the parallel port. The experiment was conducted using the *Presentation* software developed by Neurobehavioral Systems.^2^

#### 2.1.9. Design and procedure

Each set of experimental idiomatic and literal sentences were divided between two lists: the idiomatic sentence in the active voice was paired with the literal sentence in the passive voice in one list, and vice versa in the second list. In total, each list contained 260 sentences: 26 idiomatic active and 26 idiomatic passive sentences, 26 literal active and 26 literal passive sentences, and 78 literal active and 78 literal passive filler sentences. Half of all the sentences contained two arguments and the other half contained three arguments. Participants were randomly assigned one of the two lists. Each list was further subdivided into four blocks of 65 trials. Each block contained 26 experimental sentences (thirteen idiomatic and thirteen literal sentences) and 39 filler sentences. The order of the sentences was then pseudorandomized for each participant: shuffling was repeated until no more than four sentences from the same category were presented consecutively. Sixteen filler sentences (half in active voice, half in passive voice, half with two arguments, half with three arguments) were provided as practice trials before the test began. Participants were tested individually in a dimly lit lab. The viewing distance was about 70 cm from the screen. Each trial began with the presentation of a fixation cross in the center of the screen. Then, after 500 ms, the audio file started. The fixation cross remained on the screen until the audio file finished. Once the audio file ended, the relevant verb triplet was presented in a horizontal line in the middle of the screen with the three options listed, in a random order, from left to right. The verbs were shown in white text on a black background and in a sans-serif font, size 24. The verbs were displayed until the participant responded via a button press. The left button corresponded to the left-most verb, the middle button to the middle verb, and the right button to the right-most verb. Responses were made with the index finger of the dominant hand. Participants were asked to decide, as quickly as possible, which verb completed the sentence in the most meaningful way. The experiment lasted about 30 min. Breaks between the blocks were self-paced by the participants.

### 2.2. Results

The data from one participant with a bilingual background and extremely slow response times (mean = 2,887 ms) was removed so that, in total, the responses from 30 participants were included in the data analyses. Reaction times (RTs) exceeding three standard deviations from a participant’s mean were excluded (1.4% of the overall data). Mean response latencies were then calculated for those responses where participants completed the sentence with the intended verb. Idiomatic sentences were completed with the intended verb in 89% of cases, literal sentences in 80% of cases. We used R (R Core Team, 2013) and lme4 (e.g., Bates, 2005; Baayen et al., 2008) to fit a linear mixed effects model to response times. We applied a forward procedure for the model selection, starting with a minimal model and adding additional predictors only when they improved the model fit. The best model fit was determined by comparing the Akaike Information Criterion (AIC) statistics of models and a model was considered a better fit when the difference was > 2 (Sakamoto et al., 1986).

#### 2.2.1. Latency data

**FIGURE 1:**
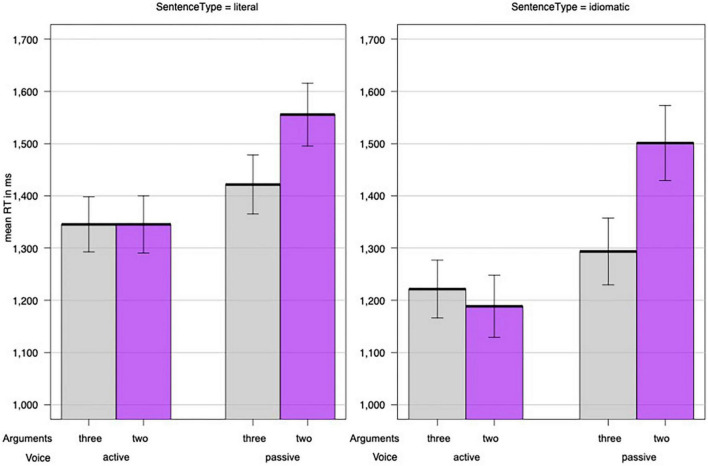
The effects of argument structure (two/three) and voice (active/passive) on idiomatic and literal sentences in Experiment 1. Y-bars represent the standard error of the mean.

In the following analyses, we treated participants and sentence pair (containing the same verb) as random effects. The fixed-effect factors of interest were Sentence Type (idiomatic/literal), Voice (active/passive), and Argument Structure (two/three). In addition, we tested the influence of the distributional variables Target Frequency (in log-transformed and centered absolute lemma frequencies) and Target Length (number of letters), as well as the confounding factors List and Block. The best model included the factors Sentence Type, Voice, Argument Structure, the interaction between Voice and Argument Structure, and Target Length. Table 3 summarizes the results. Supplementary appendix B provides the model fitted to log-transformed reaction times. Figure 1 depicts the interaction between Voice and Argument Structure.

**TABLE 3:** Fixed effects of the predictors in the linear mixed-effect model for response latencies in Experiment 1.

	Estimate (ms)	95% CI	Std. error	df	*t*-value	*p*
Intercept: literal, active, three, six letters	909	519 to 1,299	199	116		
Sentence Type (idiomatic)	−115	−219 to −12	53	100	-2.18	0.031
Voice (passive)	77	31 to 123	23	2,945	3.27	0.001
Arguments (two)	−19	−128 to 91	56	121	-0.34	0.73
Target Length (per additional letter)	53	8 to 97	23	100	2.33	0.022
Voice (passive) × arguments (two)	183	116 to 250	34	2,946	5.31	< 0.001

CI, confidence interval. The intercept refers to literal sentences in active voice with three arguments and targets with six letters.

The effect of Sentence Type indicated that, generally speaking, idiomatic sentences were processed faster than literal sentences. Most importantly, as depicted in Figure 1, both literal and idiomatic sentences were affected by Voice, indicating that the sentences in active voice were processed faster than those in passive voice. There was also an interaction between Voice and Argument Structure in that Argument Structure affected sentences in passive but not in active voice. Active sentences with two and three arguments were processed at the same faster rate. In contrast, passive sentences with three arguments were processed significantly faster than passive sentences with two arguments. The factor Target Length showed that a higher number of letters slowed target selection.

#### 2.2.2. Completion rates

In the sentence completion task, participants chose the target from the relevant verb triplet. It is important to note that all three verbs in each triplet completed the relevant sentence, whether literal or idiomatic, in a meaningful way. Therefore, as the participants could not make errors in the classical sense, we are reporting here on the “literal” completions for idiomatic sentences and will not analyze the completions for literal sentences. For idiomatic sentences, we defined the type of sentence completion as “figurative” when participants chose the verb associated with the idiom and its figurative meaning, and as “literal” when participants chose one of the other two verbs (i.e., the semantic associate or the unrelated verb).

In the following, we present our analysis of the literal (i.e., non-figurative) completions of idiomatic sentences. The completion rates were analyzed using multilevel logistic regression, with random intercepts for participants and items, and the fixed-effect factors Voice (active/passive), Argument Structure (two/three), and Target Length (number of letters). As summarized in Table 4, the best model included an interaction between the two factors Voice and Argument Structure. As in the latency data analysis, Argument Structure did not affect sentences in active voice, that is, active sentences did not differ in terms of literal completions (7 and 9%, respectively) regardless of whether they contained two or three arguments. In contrast, in passive voice, sentences containing two arguments (21%) induced significantly more literal completions than sentences containing three arguments (9%).

**TABLE 4:** Fixed effects of the predictors in the multilevel logistic regression model for the completion data of the idiomatic sentences in Experiment 1.

	Odds ratio	95% CI	*z*-value	*p*
Intercept: active, three	5.4%*	3.0 to 9.7%		
Voice (passive)	0.95	0.57 to 1.58	−0.19	0.85
Arguments (two)	0.78	0.33 to 1.84	−0.57	0.57
Voice (passive) × arguments (two)	3.42	1.66 to 7.07	3.29	0.001

*Baseline probability refers to the accuracy rate for active voice and three-argument verbs. Effects of passivation, arguments, and their interaction are expressed as odds ratios, along with their 95% confidence interval (CI).

### 2.3. Discussion

In Experiment 1, we examined whether (1) voice and (2) argument structure affected sentence processing. Our results were straightforward. First, active sentences were processed faster than passivized ones, both literal and idiomatic sentences. This result replicates previous findings (e.g., Kyriacou et al., 2020) and indicates that the effect of voice is generalized to idiomatic sentences. We will discuss the indication of this effect in more detail below, in the general discussion on whether the same principles hold for literal and idiomatic sentences (Tabossi et al., 2009)^3^. Contrary to previous findings, we observed a reversed effect of argument structure, although only in passive voice, that is, faster responses in passivized sentences with three arguments than with two arguments. The fact that this interaction between voice and argument structure was observed in both literal and idiomatic sentences suggests that idiom syntax is principled, not idiosyncratic. This finding is further corroborated by the completion data, which showed better performance (i.e., fewer literal completions) in passivized idiomatic sentences containing three arguments rather than two arguments. Considering our results, we need to reinterpret the previously observed argument structure effect (e.g., Thompson et al., 1995, 1997) differently. Previous studies examined the argument structure effect in literal sentences in active voice, while our study did not detect any argument structure effect for sentences in the active voice, whether idiomatic or literal. On the contrary, the argument structure effect occurred in both literal and idiomatic passive sentences with faster (and, in the case of idiomatic sentences, more correct) processing of sentences with three arguments than with two arguments. This finding, thus, supports our assumption that idiomatic sentences with three arguments may be perceived as more “literal” than idiomatic sentences with two arguments because they contain two literally filled arguments (rather than one). Therefore, we expected to find that passivization is less disruptive to idiomatic sentences containing three arguments than sentences containing two arguments. This prediction was borne out by the results of Experiment 1. Another explanation for the processing advantage of passive sentences with three over two arguments is argument adjacency. That is, the verb and the arguments that constitute the idiom were adjacent in the passivized sentences containing three arguments but not in the passivized sentences containing two arguments, for example, compare the three-argument sentence *Dem Jungen wurde von ihr [der Kopf] [gewaschen]* and the two-argument sentence *[Nach den Sternen] wurde von ihr [gegriffen]*. It is possible that, in order for the listener to compute the figurative meaning of a passivized idiomatic sentence, the verb and the arguments that carry the figurative meaning need to be adjacent. This difference in adjacency between sentences with two and three arguments in the passive voice may have resulted in the processing differences observed in Experiment 1. If so, argument adjacency, not argument structure, is the influential factor. We conducted Experiment 2 to test this prediction.

## 3. Experiment 2

In Experiment 2, we investigated whether argument adjacency or argument structure is more critical for the preservation of the figurative meaning of passivized idiomatic sentences. We predicted that the co-occurring constituents of an idiom are sensitive to adjacency.

In Experiment 1, we passivized all sentences in the canonical way (e.g., Burchert and de Bleser, 2010). In the canonical sentence structure of a passivized sentence with three arguments, the verb and the argument that constitutes the idiom (henceforth “the critical argument”) are adjacent, as in *Dem Jungen wurde von ihr [der Kopf] [gewaschen]*. In contrast, in the canonical structure of a passivized sentence with two arguments, the critical argument is non-adjacent to the verb, as in *[Nach den Sternen] wurde von ihr [gegriffen]*. In Experiment 1, the canonical passivization confounded the factors of argument structure and argument adjacency. Therefore, in Experiment 2, we applied non-canonical passivization in order to reverse the argument adjacency for the two- and three-argument structures. This resulted in passivized two-argument sentences in which the critical arguments were adjacent to the verb (e.g., *Von ihr wurde [nach den Sternen] [gegriffen]*) and in passivized three-argument sentences in which the critical arguments were non-adjacent to the verb (e.g., *[Der Kopf] wurde dem Jungen von ihr [gewaschen]*). This enabled us to examine the factor argument adjacency (adjacent/non-adjacent) more closely.

To date, few psycholinguistic studies have investigated the influence of constituent adjacency. In the field of verb-particle constructions (e.g., “*look up*” in “*he looked up the word*” versus “*he looked the word up*”), Gonnerman and Hayes (2005) found that shifted verb-particle structures were harder to process when long nominal phrases occurred between the verb and the particle, indicating that this type of sentence is highly sensitive to adjacency. In the field of collocations (e.g., “*provide information*” versus “*provide some of the information*”), Vilkaité (2016) showed that collocations have a general processing advantage over control phrases and that this extends to non-adjacent collocations, but that the facilitative effect of collocations might be larger for adjacent than non-adjacent collocations. Adjacency has been shown to play a role in the field of natural language processing (NLP) when it comes to the identification of idiomatic sentences. Hashimoto and Kawahara (2008) examined how ambiguous idioms are identified using two kinds of information, word sense disambiguation (WSD) features (e.g., single words in the surrounding context) and idiom-specific features (e.g., the adjacency of constituents). The results show that WSD features lead to a very high number of correct identifications (88.9%) and that adding idiom-specific features leads to even more correct identifications (89.3%). Crucially, among the idiom-specific features, adjacency was the most important factor. These findings demonstrate that constituent adjacency plays an important role in the processing of typically co-occurring instances in a language, such as verbs and their particles, and words in collocation. In our study, we extend this research to examine idiomatic sentences, in particular passivized ones. This study is, thus, the first psycholinguistic study to investigate the influence of argument adjacency on the processing of active and passive idiomatic sentences.

As mentioned above, in order to manipulate the adjacency of the arguments, we modified the syntactic structure of the sentences used in Experiment 1. Our study is the first that addresses the influence of (a) verb argument structure and (b) argument adjacency on the syntactic processing of idiomatic sentences. Specifically, we examined the processing of German idioms with either two- or three-argument verbs and manipulated the sentence structure to achieve different adjacencies of the arguments. This manipulation used canonical versus non-canonical passivization. In Experiment 1, we passivized all sentences in a canonical way such that for the passivized two-argument sentences, the argument constituting the idiom and the verb were non-adjacent. In the case of passivized three-argument sentences, the argument constituting the idiom and the verb were adjacent. In Experiment 2, we reversed these alignments in order to yield passivized two-argument sentences where the critical argument and the verb were adjacent and passivized three-argument sentences where the critical argument and the verb were non-adjacent. Note that this was possible because of the word-order variation in German. If indeed argument structure affects processing time, that is, more arguments lead to longer processing times (Shapiro et al., 1987, 1989), we should observe faster processing of two-argument sentences than three-argument sentences, irrespective of the adjacency of the arguments. If, however, the argument adjacency is the more influential factor, sentences in which the critical argument is adjacent to the verb should be processed faster than sentences in which the critical argument is non-adjacent to the verb. Such a finding would corroborate those of Experiment 1 according to which passivized sentences with three adjacent arguments showed a processing advantage over sentences with two but non-adjacent arguments. Accordingly, in Experiment 2, we should find that passivized sentences with two adjacent arguments show a processing advantage over sentences containing three non-adjacent arguments.

To summarize, we asked the same questions regarding the effects of voice and argument structure as in Experiment 1—*Is the underlying processing of active sentences comparable to that of passive sentences?*—and also expected to observe faster processing for active sentences than for passive sentences. In Experiment 2, we expanded the question of whether argument structure affects sentence processing to include the question: *Does argument adjacency (i.e., the adjacency of the critical argument and the verb) affect sentence processing in general and that of idiomatic sentences in particular?* If argument structure affects the preservation of the figurative meaning in passivized sentences, the results of Experiment 1 (faster processing of three-argument sentences over two-argument sentences) will be replicated. If, however, argument adjacency exerts a greater influence on the preservation of the figurative meaning of passive sentences, passivized idiomatic sentences with critical arguments adjacent to the verb (i.e., two-argument sentences in Experiment 2) will show a processing advantage over those with critical arguments non-adjacent to the verb (i.e., three-argument sentences in Experiment 2).

### 3.1. Materials and methods

#### 3.1.1. Participants

Thirty students (6 male, 24 female, mean age of 22.9 years) from the University of Konstanz who did not take part in Experiment 1 were paid five euros for their participation. All were native speakers of German. No participants were dyslexic and all had normal or corrected-to-normal vision.

#### 3.1.2. Materials

Supplementary appendix C lists all the stimuli used in Experiment 2. The stimulus materials were the same as in Experiment 1, with a few modifications. First, the sentences in passive voice had a different argument adjacency. The passive sentences with two arguments from Experiment 1, in which the critical argument was non-adjacent to the verb (“*[Nach den Sternen] wurde immer von ihr [gegriffen]*”), were given an adjacent configuration in Experiment 2 (“*Von ihr wurde immer [nach den Sternen] [gegriffen]*”). Similarly, the passive sentences with three arguments from Experiment 1, in which the critical argument was adjacent to the verb (“*Dem Jungen wurde von ihr [der Kopf] [gewaschen]*”), were given a non-adjacent configuration in Experiment 2 (“*[Der Kopf] wurde dem Jungen von ihr [gewaschen]*”). Second, the sentences in active voice differed in their adjacency. All active sentences were transformed from an unmarked structure in which the idiomatic arguments were adjacent to the verb into a marked structure with a topicalized object, resulting in a structure where the critical arguments were non-adjacent to the verb. Thus, all sentences featured an unmarked sentence structure in Experiment 1 and a marked sentence structure in Experiment 2. It is possible that idioms with a structure in which the critical argument is not adjacent to the verb are more strenuous for working memory than adjacent configurations are. As this effect may be more pronounced in auditory than in visual presentation mode, we changed the modality of the sentence presentation from auditory in Experiment 1 to visual in Experiment 2. Table 5 lists all eight conditions of Experiments 1 and 2.

**TABLE 5:** Comparison of argument adjacency in Idiomatic and Literal sentence pairs across Experiments 1 and 2.

	Idiomatic	Literal
**Experiment 1**		
**Two**		
Active	Sie hat immer [nach den Sternen] [gegriffen].	Das Mädchen hat [nach den Bonbons] [gegriffen].
Passive	[Nach den Sternen] wurde immer von ihr [gegriffen].	[Nach den Bonbons] wurde von dem Mädchen [gegriffen].
**Three**		
Active	Sie hat dem Jungen [den Kopf] [gewaschen].	Er hat der Tochter [die Haare] [gewaschen].
Passive	Dem Jungen wurde von ihr [der Kopf] [gewaschen].	Der Tochter wurden von ihm [die Haare] [gewaschen].
**Experiment 2**		
**Two**		
Active	[Nach den Sternen] hat sie immer [gegriffen].	[Nach den Bonbons] hat das Mädchen [gegriffen].
Passive	Von ihr wurde immer [nach den Sternen] [gegriffen].	Von dem Mädchen wurde [nach den Bonbons] [gegriffen].
**Three**		
Active	[Den Kopf] hat sie dem Jungen [gewaschen].	[Die Haare] hat er der Tochter [gewaschen].
Passive	[Der Kopf] wurde dem Jungen von ihr [gewaschen].	[Die Haare] wurden der Tochter von ihm [gewaschen].

The critical (idiomatic) arguments of the verb are in brackets.

The sentence structures of the literal sentences and the filler sentences were adapted to match the structure of the idiomatic sentences. Other than these changes in sentence structure, all sentences and verb targets, as well as the proportion of experimental and filler sentences were the same as in Experiment 1.

#### 3.1.3. Apparatus

The stimuli were presented on a 17′′ flat-screen monitor (Flatron L1810B) connected to a BEST personal computer. Response latencies were recorded via a key press on a three-button box connected to the parallel port. The experiment was conducted using the *Presentation* software developed by Neurobehavioral Systems.^4^

#### 3.1.4. Procedure

Each trial began with the presentation of a fixation cross in the center of the screen for 500 ms. The fixation cross was then replaced by the words of a sentence, presented in the middle of the screen, with the fourth (i.e., the middle) word aligned with the center of the screen. The sentence remained on the screen until the participant responded via a button press, after which the verb triplet was presented in horizontal alignment, in a central position, and with the three options listed in random order from left to right. The sentence and the verbs were shown in white text on a black background in a sans-serif font, size 26 and 24, respectively. All other experimental conditions were the same as in Experiment 1.

### 3.2. Results

The responses from one participant with extremely high non-figurative verb completions (> 50%) were removed, meaning a total of 29 participants were included in the data analyses. Reaction times (RTs) exceeding three standard deviations from a participant’s mean were excluded (1.3% of the overall data). Mean response latencies were calculated for the responses in which participants completed the sentence with the “figurative” verb, as was the case in 87% of idiomatic sentences and 77% of literal sentences. Note that all verb completions resulted in meaningful sentences, meaning there was no classical error count. As in Experiment 1, we performed linear mixed effects analysis to response times using R (R Core Team, 2013) and lme4 (e.g., Bates, 2005; Baayen et al., 2008) and applied a forward procedure for the model selection. The best model fit was obtained by comparing the AIC statistics between models as in Experiment 1 (Sakamoto et al., 1986).

#### 3.2.1. Latency data

**FIGURE 2:**
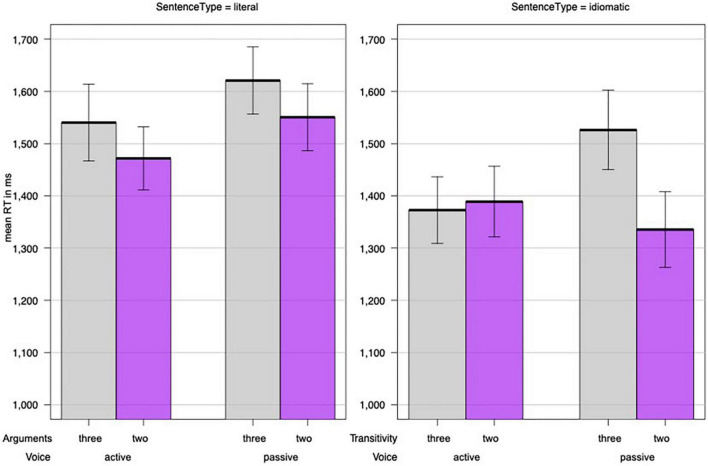
The effects of argument structure (two/three) and voice (active/passive) on idiomatic and literal sentences in Experiment 2. Y-bars represent the standard error of the mean.

As in Experiment 1, we treated participants and sentence pair (containing the same verb) as random effects and examined the fixed-effect factors Sentence Type (idiomatic/literal), Voice (active/passive), and Argument Structure (two/three). In addition, we tested the influence of Target Frequency (in log-transformed and centered absolute lemma frequencies), Target Length, List, and Block. As in Experiment 1, the best model included the factors Sentence Type, Voice, Argument Structure, and Target Length, as well as interaction between Voice and Argument Structure and a three-way interaction of Sentence Type, Voice, and Argument Structure. Table 6 summarizes the model fit to raw reaction times, Supplementary appendix D provides the model fit to log-transformed reaction times, and Figure 2 depicts the interaction. As in Experiment 1, idiomatic sentences were processed (marginally) faster than literal ones and sentences in the active voice were processed faster than those in the passive voice. Again, the results for Target Length demonstrated a higher number of letters prolonged the target selection. The significant three-way interaction indicated the following: as in Experiment 1, neither the literal nor idiomatic sentences in the active voice were affected by Argument Structure and there was equivalent processing of both two- and three-argument sentences. In contrast to Experiment 1, literal sentences in passive voice also remained unaffected by argument structure. Furthermore, idiomatic sentences in passive voice displayed the opposite effect of Argument Structure found in Experiment 1 in that sentences containing two arguments were processed significantly faster than sentences containing three arguments.

**TABLE 6:** Fixed effects of the predictors in the linear mixed-effect model for response latencies in Experiment 2.

	Estimate (ms)	95% CI	Std. Error	df	*t*-value	*p*
Intercept: literal, active, three, six letters	963	510 to 1,416	231	108	4,17	< 0.0001
Sentence (idiomatic)	−168	−341 to 6	88	117	−1.9	0.0605
Voice (passive)	80	10 to 150	36	2,828	2.25	0.0248
Arguments (two)	−78	−259 to 102	92	117	−0.85	0.3970
Target Length (per additional letter)	71	19 to 123	27	98	2.67	0.0091
Sentence (i) × voice (p)	72	−26 to 171	50	2,828	1.44	0.1509
Sentence (i) × arguments (two)	87	−167 to 342	130	117	0.67	0.5028
Voice (p) × arguments (two)	1	−102 to 103	52	2,828	0.02	0.9883
Sentence (i) × voice (p) × Arguments (two)	−210	−355 to −65	74	2,828	−2.84	0.0046

CI, confidence interval. The intercept refers to literal sentences in active voice with three arguments and targets with six letters.

#### 3.2.2. Completion rates

As in Experiment 1, we defined the type of sentence completion as “figurative” when participants chose the verb that completed the sentence figuratively and as “literal” when participants chose one of the verbs that completed the sentence literally. The following report again focuses on the literal completions of idiomatic sentences. As in Experiment 1, the completion rates were analyzed using multilevel logistic regression, with random intercepts for participants and items, and the fixed-effect factors Voice (active/passive), Argument Structure (two/three), and Target Length (number of letters). As summarized in Table 7, Voice alone had a significant effect. Nevertheless, as expected, the mean completion rates point to a reversed pattern as the one found in Experiment 1, with more literal completions in the passivized sentences containing three arguments (17%) than in the passivized sentences containing two arguments (9%), and no difference between active sentences regardless of whether they contained two or three arguments (9 and 12%, respectively).

**TABLE 7:** Fixed effects of the predictors in the multilevel logistic regression model for the completion data of the idiomatic sentences in Experiment 2.

	Odds ratio	95% CI	*z*-value	*p*
Intercept: active, three	7.7%*	4.5 to 12.8%		
Voice (passive)	1.62	1.04 to 2.51	2.13	0.033
Arguments (two)	0.71	0.31 to 1.64	-0.80	0.43
Voice (passive) × arguments (two)	0.58	0.28 to 1.19	-1.48	0.14

*Baseline probability refers to the accuracy rate for active voice and three-argument verbs. Effects of passivization, arguments, and their interaction are expressed as odds ratios, along with their 95% confidence interval.

### 3.3. Discussion

In Experiment 2, we investigated whether the adjacency of the critical arguments and the verb affects the preservation of the figurative meaning in passivized idiomatic sentences. To this end, we transformed the sentences of Experiment 1 in such a way that the passivized two-argument sentences in which the critical argument is non-adjacent to the main verb from Experiment 1 became adjacent in Experiment 2, and vice versa for the passivized three-argument sentences. With regard to the main effects, we replicated the findings of Experiment 1. Sentences in the active voice had a processing advantage over sentences in the passive voice and this result was observed for both literal and idiomatic sentences. Note that all active and passive sentences were non-canonical in Experiment 2 and thus did not differ in this respect. Crucially, the direction of the interaction between Voice and Argument Structure, whereby passivized sentences containing three arguments had a processing advantage over passivized sentences containing two arguments in Experiment 1, did not replicate. On the contrary, in Experiment 2, idiomatic passivized sentences containing two arguments displayed a processing advantage over sentences containing three arguments. The completion rates further supported this result as the passivized sentences containing two arguments in which the idiomatic arguments were adjacent to the verb had a processing advantage over the passivized sentences containing three arguments in which the idiomatic arguments were non-adjacent to the verb. Given that, in Experiment 2, passivized idioms containing two arguments had an adjacent argument-verb configuration and passivized idioms containing three arguments had a non-adjacent argument-verb configuration, this finding supports our hypothesis that the adjacency of the arguments and the verb that constitute the idiom affects the preservation of the figurative meaning in passivized sentences.

#### 3.3.1. *Post hoc* analysis: argument structure versus argument adjacency

**FIGURE 3:**
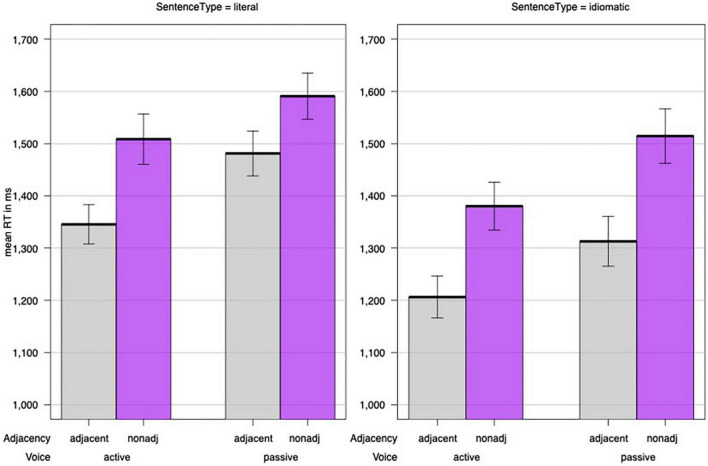
The effect of argument adjacency on idiomatic and literal sentences for Experiments 1 and 2. Y-bars represent the standard error of the mean.

To examine whether argument adjacency may be a better predictor for determining idiomatic processing than Argument Structure we conducted the following *post hoc* analyses for the combined data from Experiments 1 and 2. To examine the influence of adjacency, we coded sentences as Adjacent when their verb and critical argument were adjacent. That is, the following sentences were coded as adjacent: (a) all active sentences (containing two or three arguments) from Experiment 1, (b) the passive sentences containing three arguments from Experiment 1, and (c) the passive sentences containing two arguments from Experiment 2. The remaining sentences were coded as non-adjacent including (a) all active sentences (containing two and three arguments) from Experiment 2, (b) the passive sentences containing three arguments from Experiment 2, and (c) the passive sentences containing two arguments from Experiment 1. We then used R and lme4 to fit linear mixed effects models to reaction times. As in Experiments 1 and 2, we treated participants and sentence pair (containing the same verb) as random effects and included the fixed effects Sentence Type (idiomatic/literal), Voice (active/passive), and Target Length (number of letters). We then compared, using the AIC, whether the inclusion of the factor Argument Structure (two/three) or argument adjacency (adjacent/non-adjacent) better improved the model. As indicated in Table 8 (see Supplementary appendix E for log-transformed reaction times), the factor argument adjacency provided a better model fit and confirmed that non-adjacent constituents slowed responses to idiomatic sentences. Figure 3 depicts this effect of adjacency.

**TABLE 8:** Fixed effects of the predictors in the linear mixed-effect model for response latencies in the *post hoc* analysis.

	Estimate (ms)	Std. error	df	*t*-value	*p*	AIC
**Analysis with adjacency**						**92,614**
Intercept	883	150	230	5.87	< 0.0001	
Sentence Type (idiomatic)	−182	44	275	−4.13	< 0.0001	
Voice (passive)	114	13	5,776	9.09	< 0.0001	
Adjacency (non-adjacent)	72	25	5,714	2.89	0.0039	
Target Length (per additional letter)	61	17	203	3.56	0.0005	
Sentence Type (i) × Adjacency (non-adj)	109	35	5,249	3.12	0.0018	
**Analysis with arguments**						**92,679**
Intercept	920	152	226	6.07	< 0.0001	
Sentence Type (idiomatic)	−127	41	202	−3.12	0.0021	
Voice (passive)	116	13	5,778	9.14	< 0.0001	
Arguments (two)	−7	41	202	−0.17	0.8637	
Target Length (per additional letter)	62	17	202	3.53	0.0005	

The intercept in the analysis with adjacency refers to literal sentences in active voice with adjacent arguments and targets with six letters; the intercept in the analysis with arguments refers to literal sentences in active voice with three arguments and targets with six letters.

The *post hoc* analysis confirmed the effect of argument adjacency, in that idiomatic sentences with adjacent arguments were processed faster than idiomatic sentences with non-adjacent arguments. Thus, the results of Experiment 2 and the *post hoc* analysis confirm the hypothesis that argument adjacency rather than argument structure determines whether passivized idiomatic sentences preserve their figurative meaning. Furthermore, the *post hoc* analysis included not only passive but also active sentences with adjacent vs. non-adjacent arguments (due to topicalization). We may, thus, assume that argument adjacency affects both passive and active sentences, even though its effect on the latter needs to be studied further in future research.

One may wonder whether the different presentation modes—auditory in Experiment 1 and visual in Experiment 2—affected the results. However, as the main effects of Sentence Type (idiomatic vs. literal) and Voice (active vs. passive) were replicated in Experiment 2, we may assume that the different presentation modes did not affect our results. Further support for this assumption is provided by Titone and Libben (2014:489) who argue that it is unclear whether there are any systematic differences between visually and auditorily presented materials in the absence of any overt prosodic manipulation.

## 4. General discussion

In the present study, we conducted two sentence completion experiments in which participants decided which of three verbs was the best option to complete a sentence. In Experiment 1, we examined whether (1) voice and (2) argument structure affected sentence processing. In Experiment 2 and in the *post hoc* analysis, we examined whether (1) voice and (2) adjacency of the verb to the critical argument affected sentence processing. We examined the processing of both idiomatic and literal sentences and compared the processing of active versus passive sentences. Furthermore, we asked whether the argument structure and argument adjacency affect the processing of idiomatic and literal sentences. Overall, we focused on two core questions. Question 1: Are sentences in the active voice, in particular idiomatic sentences, processed differently from sentences in the passive voice? Question 2: Does argument structure (i.e., containing two or three arguments) or argument adjacency (i.e., the adjacency of the verb to its arguments) affect sentence processing in general and that of idiomatic sentences in particular? We interpret our results as showing that the answer to Question 1 is “yes” and that the answer to Question 2 is “yes, argument adjacency does”. We elaborate further on these questions below.

Question 1 asked whether passive sentences are processed differently from active ones and whether this effect is generalized to idiomatic sentences. Our results were straightforward. Active sentences were processed faster than passivized ones and this effect was observed for both idiomatic and literal sentences. Our findings converge with the results of previous studies based on eye tracking (Kyriacou et al., 2020). The completion rates further supported this finding for idiomatic sentences. That is, the effect of voice was found to be generalized to idiomatic sentences, indicating that the same principles hold for both literal and idiomatic sentences (Tabossi et al., 2009). We interpret these results as corroborating evidence for the claim that the syntactic processing of idiomatic sentences has the same underlying principles as the syntactic processing of literal sentences.

Question 2 asked whether argument structure (i.e., containing two or three arguments) or argument adjacency (i.e., the adjacency of the critical argument to the verb) affects sentence processing in general and idiomatic sentences in particular. In Experiment 1, we found that argument structure had an effect on both idiomatic and literal sentences in that sentences with three arguments in passive voice were processed faster than sentences with two arguments in passive voice. However, passivized sentences containing two arguments had a different adjacency of their critical argument to their verb than the passivized sentences containing three arguments. Therefore, in Experiment 2, we swapped the sentence structure of all sentences and investigated whether the adjacency of the verb to its arguments affects the passivizability of idiomatic sentences. This time, the passivized two-argument sentences with critical arguments adjacent to the verb had a processing advantage over the passivized three-argument sentences with critical arguments that were non-adjacent to the verb. Furthermore, the *post hoc* analysis also supported the finding that argument adjacency rather than argument structure affects sentence processing, in particular that of idiomatic sentences. A first glance at the results of Experiment 2 may point to an asymmetry of the adjacency effect in that active sentences featuring non-adjacent idiomatic constituents (i.e., idiomatic active sentences with two and three arguments) are processed equally fast as passive sentences featuring adjacent constituents (i.e., corresponding to idiomatic passive sentences with two arguments). One could argue that these results indicate some sort of asymmetry in that adjacency seems crucial for passive sentences with idiomatic constituents but not for active sentences with idiomatic constituents. The source of that might be the different orderings of the arguments, licensing different logic relations independent of the syntactic structure (see Mondal, 2020, 2022 for meaning relations). In that sense, the difference between active and passive sentences in terms of adjacency may arise not only due to syntactic relations but also due to different meaning relations and information processing capacities.

We favor a different explanation, though, which relates to the interaction between Sentence Type, Voice, and transitivity (in Experiment 2). First, all active (idiomatic) sentences in Experiment 2 feature non-adjacent constituents, so that the effect of adjacency can materialize for passive sentences only. Second, the effect of voice is a strong and prevailing effect (see Tannenbaum and Williams, 1968; Olson and Filby, 1972; Kyriacou et al., 2020) with a general processing advantage of active (idiomatic) sentences over passive ones. Importantly, this effect of voice is not overridden by adjacency (see main effects of Voice and Figure 2). Under this view, we anticipate similar responses to active sentences with non-adjacent (idiomatic) constituents and passive sentences with adjacent (idiomatic) constituents. This assumption is further confirmed by the *post hoc* analysis (see also Figure 3), indicating that adjacency affects both active and passive sentences with idiomatic constituents. In the following, we consider the compatibility of our findings with current models of idiom processing.

### 4.1. Discussion of models on idiom processing

It is still an open question as to whether our results are compatible with linguistic theories, such as construction grammar. As a construction, an idiom has a unique syntax (see Fillmore et al., 1988; Croft and Cruse, 2004) and, therefore, differs from literal language. However, if we assume that language falls on a continuum with substantive idioms (all elements of the idiom are fixed) at one end, schematic idioms (the elements of the idiom that are lexically open) and strong collocations in the middle, and less collocated literal language at the other end, idioms and literal sentences should be seen as constructions and should, thus, behave in similar ways. However, a psycholinguistic implementation of this topic is beyond the scope of this study. Instead, what we have shown in this study is that the sentence’s verb (be it literal or idiomatic) and, in particular, the configuration of the verb and its arguments, provides information that is critical for sentence processing. While the number of arguments is not crucial when it comes to the interpretation of syntactically modified idioms, their configuration and, specifically, their adjacency to the verb strongly affect the recognition of the figurative meaning in modified idiomatic sentences.

In relation to psycholinguistic models, non-compositional approaches, including the *idiom list* hypothesis (Bobrow and Bell, 1973), the *lexical representation* hypothesis (Swinney and Cutler, 1979), and the *direct access* hypothesis (Gibbs, 1980), assume a fixed entry that represents the figurative meaning. As a consequence, neither argument structure nor argument adjacency are expected to play a role in these accounts. Furthermore, according to the *direct access* hypothesis, listeners access the conventional, idiomatic meaning of an utterance before they decide whether the literal meaning is appropriate (Gibbs, 1980). Therefore, idiomatic sentences are expected to have a processing advantage over literal sentences. Indeed, this was the case in our study, where idiomatic sentences were processed faster than literal ones. However, the collocations of the idiomatic sentences in our study were also stronger than in the literal sentences, as is reflected in the higher sentence-completion rates for idiomatic (76%) than for literal sentences (52%). Therefore, the processing advantage of idiomatic over literal sentences in our study may not serve as an indication as to whether idioms are stored holistically and accessed directly. In a previous study that rigorously matched this collocation strength in idiomatic and literal sentences, sentence completion rates were comparable and processing speeds for figurative and literal meanings were equally fast (Smolka et al., 2007a).

According to Cacciari and Tabossi’s (1988) configuration hypothesis, which is a hybrid model, a sentence is understood figuratively as soon as the idiom is recognized. The point at which it is recognized as an idiom is referred to as the “idiom key.” According to these authors, a sentence in which the idiom key occurs earlier will be processed faster than a sentence in which the idiom key occurs later. Regardless of whether the sentence contains two or three arguments, the direct object plays a vital role in constituting the figurative meaning of idiomatic sentences. In fact, in some idioms, the direct object is the idiom key (e.g., *[den Kopf]* in “*jemandem [den Kopf] waschen*”; F: “*to give someone [a piece of his/her mind]*”). Thus, according to the configuration hypothesis, passivized idiomatic sentences that feature this critical argument in a sentence-initial position should be processed faster (e.g., “*[Der Kopf] wurde dem Jungen von ihr gewaschen*”) than those in which this critical argument occurs in a sentence-medial position (e.g., “*Dem Jungen wurde von ihr [der Kopf] gewaschen*”). However, our findings from Experiment 1 showed that sentences with an early idiom key (e.g., “*[Der Kopf] wurde dem Jungen von ihr gewaschen*”) exhibited the highest processing costs of all idiomatic sentences, a finding that contradicts the configuration hypothesis.

According to the superlemma theory (Sprenger et al., 2006), the syntactic restrictions of individual idioms are captured in the superlemma entry for each idiom, independently of other factors such as compositionality. If syntactic restrictions are truly independent of other factors, we would expect to find no effects from either argument structure or argument adjacency. However, we did observe effects from both argument structure and argument adjacency in both Experiments 1 and 2, a result that cannot be accounted for by the predictions of the superlemma theory. Furthermore, as pointed out in previous studies (e.g., Tabossi et al., 2009; Holsinger, 2013), the restrictions associated with the superlemma should always apply without exceptions and without variations in degree. That is, if the superlemma informs us that an idiom such as “*to reach for the stars*” cannot be passivized, the idiom should be unacceptable in any passivized form, regardless of whether it is in a canonical form or not. However, in our study, participants did not reject passivized idioms in general, only those sentences in which the idiomatic arguments were not adjacent to the verb. Therefore, our findings do not support the superlemma theory.

Our results seem compatible with the constraint-based model of idiom processing presented by Libben and Titone (2008), albeit with some small adaptations. According to this model, readers or listeners generate simultaneous activations for the semantic representation of the literal interpretation and the figurative representation of the idiom. The figurative activation increases over time as more of the idiomatic configuration is revealed and, as soon as the final word of the idiom is encountered, both the idiomatic meaning and products of a literal compositional analysis of the string become available. A variety of factors modulate the comprehension from the start, and all sources of information interact in a time-dependent fashion (Libben and Titone, 2008:1,116), including the factors familiarity (which has an early influence), literal plausibility, word frequency, and compositionality (which has a late influence). As people make use of several relevant sources of information in idiom comprehension, the factor adjacency could be easily integrated in this model. Furthermore, as the constraint-based model is solely based on the comprehension of verb-*x*-noun idioms (e.g., *kick the bucket*, *take a beating*) and is, thus, limited to the comprehension of idioms with two arguments, the theory would also need to be extended to cover idioms with three arguments.

The stem-based frequency account (Smolka et al., 2007a,b; Rabanus et al., 2008; Smolka and Eulitz, 2020) assumes that idiom processing resembles literal processing and, therefore, makes no specific predictions about the syntactic processing or the syntactic restrictions of idiomatic phrases. According to this account, a combination of word constituents—for instance “*reach*” and “*stars*”—activates the concept that expresses the figurative meaning “*to try to achieve something impossible*”. At the concept level, the meaning behind this concept then activates a closely related concept, *ambition*. Importantly, the frequency of a particular word combination (*reach for* + *stars*) determines how strongly the common concept will be activated. At this point, it is useful to elaborate on the term “word combination,” an expression that does not specify how close the combination’s constituents need to be. For instance, one syntactic operation that is often used in studies on the syntactic flexibility of idioms is adverb insertion, which is widely recognized as the most easily accepted manipulation (see Gibbs and Nayak, 1989; Tabossi et al., 2008, 2009). For example, the Italian idiom “*toccare il fondo*” (L: *touch the bottom*; F: “*reach rock bottom*”) can be turned into “*toccare purtroppo il fondo*” (L: *touch unfortunately the bottom*) without losing its figurative meaning (Tabossi et al., 2009). The constituents of the idiom occur in combination, but there is a greater distance between them due to the adverb insertion. The question is, how large can this distance be before losing the figurative meaning? According to the results of studies on adverb insertion, a certain distance between the constituents of an idiomatic combination is possible. However, the results of the present study indicate that this distance cannot be arbitrarily large. Consequently, information about the distance between idiomatic arguments (i.e., the adjacency of arguments) needs to be integrated into the stem-based frequency account. The results of our study suggest that when fewer non-idiomatic arguments intervene between idiomatic arguments (in other words, the closer the idiomatic arguments are), the more likely it is that the figurative meaning will be retained.

According to Smolka and colleagues, there is no difference in how idiomatic and literal sentences are processed and both types of sentences can be combined in one model. Indeed, in Experiment 1, we found that the processing of the idiomatic and literal sentences did not differ regarding either the main effect of voice (active sentences were processed faster than passive sentences) or regarding the interaction between voice and argument structure (sentences containing three arguments were processed faster than sentences containing two arguments when both sentences were in passive voice but not when they were in active voice). In Experiment 2, the main effect of voice was, again, observed for both idiomatic and literal sentences (active sentences were processed faster than passive sentences). However, the interaction between voice and argument structure in Experiment 2 (sentences containing two arguments were processed faster than sentences containing three arguments in passive voice, but not in active voice) was observed for idiomatic sentences only. This suggests that idiomatic sentences are more sensitive to the adjacency constraint than literal sentences are, probably because of the strong collocations associated with idioms. Although there are many collocations in literal language as well, the collocations of the idiomatic sentences in our study seemed to be stronger, as is reflected by the higher sentence-completion rates for idiomatic sentences (76%) compared to literal sentences (52%). Therefore, our findings support the notion that the adjacency of co-occurring arguments plays an important role. The figurative meaning is preserved in syntactically modified idiomatic sentences as long as the verb and critical idiomatic argument are adjacent. This adjacency constraint holds true, at least for German, a language with a very flexible sentence structure. This is important to note because we do agree with Bargmann and Sailer (2018) who claim that differences in the syntactic constructions of a language have far-reaching consequences for the flexibility of multiword expressions. Therefore, we presume that the syntactic properties of idioms reflect the syntactic peculiarities of the language in question, with the result that the rendering of German idioms is syntactically flexible as long as the adjacency constraint is satisfied.

## 5. Conclusion

We explored the fundamental question of whether literal and idiomatic sentence processing is similar under the influence of syntactic transformations focusing on two factors that have not been addressed thus far: argument structure (i.e., two and three arguments) and argument adjacency (i.e., the adjacency between the verb and the idiomatic arguments). We found that both literal and idiomatic sentences are equally affected by passivization and argument structure, indicating they are processed using the same underlying mechanisms. However, our second experiment demonstrated that argument adjacency is more important for the retention of the figurative meaning than argument structure. Furthermore, the adjacency effect was more prominent for idiomatic than literal sentences, indicating that the critical arguments are more strongly collocated in idiomatic sentences than they are in literal sentences. Although it remains necessary to study whether these effects are present in languages other than German, we have taken the first steps toward a better understanding of idiom processing by demonstrating that the effect of argument adjacency overrules that of argument structure in syntactically transformed idioms in German.

## Data availability statement

The original contributions presented in the study are included in the article/Supplementary material, further inquiries can be directed to the corresponding author/s.

## Ethics statement

Ethical review and approval was not required for the study on human participants in accordance with the local legislation and institutional requirements. The patients/participants provided their written informed consent to participate in this study.

## Author contributions

LR and ES contributed to the conception, design of the study, and performed the statistical analysis. LR organized the materials, conducted the pre-tests and experiments, and wrote the first draft of the manuscript. Both authors contributed to the manuscript submission, revision and approved the submitted version.

## Footnotes

1 Arguments are presented in brackets.

2
http://www.neurobs.com/

3 Given that sentence completion rates were higher for idiomatic than for literal sentences, which may have resulted in faster responses to idiomatic than to literal sentences, we do not consider this finding relevant to the theoretical models. However, it would be possible to draw subsets of literal and idiomatic sentences with similar sentence completion rates (in active voice) and compare the responses.

4
http://www.nbs.neuro-bs.com
