# Supplementary material for: The wrong horse was bet on: the effects of argument structure versus argument adjacency on the processing of idiomatic sentences
Source: Front Psychol. 2023 May 4;14:1123917. doi: 10.3389/fpsyg.2023.1123917 (PMC10194116; doi:10.3389/fpsyg.2023.1123917)
Supplement: Supplementary file 1 [file Table_1.DOCX]

**Appendix A**

*Experimental Sentences in Active and Passive Voice of Experiment 1*

|  | **Two-Argument Sentences – Active Voice** | | | | |
| --- | --- | --- | --- | --- | --- |
| **Sentence Pair** | **Sentence Type** | **Sentence** | **Target Verb** | | |
|  |  |  | **Figurative** | **Semantic** | **Unrelated** |
| 1 | Idiomatic | Er hat in den sauren Apfel…  (L) He has *bitten* into the sour apple.  (F) He has swallowed the bitter pill. | *gebissen*  (‘bitten’) | *gespuckt*  (‘spat’) | *geschnitten*  (‘cut’) |
|  | Literal | Der Hund hat den Postboten bereits…  (L) The dog has already bitten the postman. | *gebissen*  (‘bitten’) | *gezwickt*  (‘pinched’) | *gerochen*  (‘smelled’) |
| 2 | Idiomatic | Sie haben beide ins gleiche Horn…  (L) They have both *blown* into the same horn.  (F) They have both sung from the same hymn sheet. | *geblasen*  (‘blown’) | *gehaucht*  (‘aspirated’) | *geseufzt*  (‘sighed’) |
|  | Literal | Der talentierte Musiker hat das Saxophon…  (L) The talented musician has played the saxophone. | *geblasen*  (‘blown’) | *gepustet*  (‘puffed’) | *geputzt*  (‘cleaned’) |
| 3 | Idiomatic | Die Jungs haben gemeinsam die Schulbank…  (L) The boys have *squeezed* the school desk together.  (F) The boys have been to school together. | *gedrückt*  (‘squeezed’) | *gepresst*  (‘pressed’) | *genutzt*  (‘used’) |
|  | Literal | Sie hat alle ihre Pickel wund…  (L) She has squeezed all her pimples sore. | *gedrückt*  (‘squeezed’) | *gekratzt*  (‘scratched’) | *gelegen*  (‘couched’) |
| 4 | Idiomatic | Sie hat immer nach den Sternen…  (L) She has always *grasped* at the stars.  (F) She has always reached for the stars. | *gegriffen*  (‘grasped’) | *gelangt*  (‘grabbed’) | *gelebt*  (‘lived’) |
|  | Literal | Das Mädchen hat nach den Bonbons…  (L) The girl has grasped at the candy. | *gegriffen*  (‘grasped’) | *gelangt*  (‘grabbed’) | *getastet*  (‘fumbled’) |
| 5 | Idiomatic | Der Junge hat schnell den Schnabel…  (L) The boy has quickly *held* the spout.  (F) The boy has held his tongue quickly. | *gehalten*  (‘held’) | *gestützt*  (‘supported’) | *geschüttelt*  (‘shaken’) |
|  | Literal | Ich habe die Hundeleine sehr kurz…  (L) I have held the leash very shortly. | *gehalten*  (‘held’) | *gefasst*  (‘grabbed’) | *gebraucht*  (‘needed’) |
| 6 | Idiomatic | Er hat lange an der Strippe…  (L) He has *hung* on the strap a long time.  (F) He has been on the phone a long time. | *gehangen*  (‘hung’) | *gependelt*  (‘swung’) | *gesprochen*  (‘spoken’) |
|  | Literal | Der Regenschirm hat lange am Haken…  (L) The umbrella has been hanging on the hook  for a long time. | *gehangen*  (‘hung’) | *gependelt*  (‘swung’) | *gewackelt*  (‘shaken’) |
| 7 | Idiomatic | Er hat ordentlich auf den Putz…  (L) He has fairly *beaten* on the plastering.  (F) He has fairly kicked up his heels. | *gehauen*  (‘beaten’) | *gehackt*  (‘chopped’) | *gestrichen*  (‘painted’) |
|  | Literal | Er hat kräftig auf den Tisch…  (L) He has strongly thumped onto the table. | *gehauen*  (‘beaten’) | *getrommelt*  (‘drummed’) | *gemurmelt*  (‘mumbled’) |
| 8 | Idiomatic | Das junge Mädchen hat nach Blut…  (L) The young girl has *drooled* over blood.  (F) The young girl has got thirst for blood. | *gelechzt*  (‘drooled’) | *gegiert*  (‘lusted for’) | *geschielt*  (‘squinted’) |
|  | Literal | Der durstige Marathonläufer hat nach Wasser…  (L) The thirsty marathon runner has drooled over water. | *gelechzt*  (‘drooled’) | *gegiert*  (‘lusted for’) | *gewühlt*  (‘nuzzled’) |
| 9 | Idiomatic | Die Frau hat endlich den Schleier…  (L) The woman has finally *lifted* the veil.  (F) The woman has finally lifted the curtain. | *gelüftet*  (‘lifted’) | *gerafft*  (‘gathered’) | *gestreift*  (‘roamed’) |
|  | Literal | Sie hat nie das miefende Zimmer…  (L) She has never ventilated the stinking room. | *gelüftet*  (‘lifted’) | *getrocknet*  (‘dried’) | *gemietet*  (‘rented’) |
| 10 | Idiomatic | Sie hat den Mund zu voll…  (L) She has *taken* the mouth too full.  (F) She has bitten off more than she could chew. | *genommen*  (‘taken’) | *getan*  (‘done’) | *gelassen*  (‘let’) |
|  | Literal | Die abhängige Frau hat die Tabletten…  (L) The addicted woman has taken the pills. | *genommen*  (‘taken’) | *gefordert*  (‘requested’) | *gelassen*  (‘let’) |
| 11 | Idiomatic | Sie hat nach Tagen das Feld…  (L) She has *evacuated* the field after days.  (F) She has beaten a retreat after days. | *geräumt*  (‘evacuated’) | *geleert*  (‘emptied’) | *gekehrt*  (‘swept’) |
|  | Literal | Das Mädchen hat letztendlich dasZimmer…  (L) The girl has eventually cleared out the room. | *geräumt*  (‘evacuated’) | *gewechselt*  (‘changed’) | *gemeint*  (‘meant’) |
| 12 | Idiomatic | Der Teufel hat das kleine Kind…  (L) The devil has *ridden* the little child.  (F) The devil has gotten into the little child. | *geritten*  (‘ridden’) | *gejagt*  (‘hunted’) | *geärgert*  (‘annoyed’) |
|  | Literal | Sie hat täglich das junge Pferd…  (L) She has ridden the young horse daily. | *geritten*  (‘ridden’) | *gezügelt*  (‘bitted’) | *gemocht*  (‘liked’) |
| 13 | Idiomatic | Die junge Frau hat mächtig Schaum…  (L) The young woman has *beaten* plenty of foam.  (F) The woman has spoken big. | *geschlagen*  (‘beaten’) | *geprügelt*  (‘basted’) | *gebildet*  (‘built’) |
|  | Literal | Sie hat ihn aus purer Notwehr…  (L) She has beaten him out of pure self-defense | *geschlagen*  (‘beaten’) | *geohrfeigt*  (‘slapped’) | *geschützt*  (‘protected’) |
| 14 | Idiomatic | Er hat ganz alleine den Laden…  (L) He has *worked* the shop by himself.  (F) He has run the show by himself. | *geschmissen*  (‘thrown’) | *geschwungen*  (‘swung’) | *gezahlt*  (‘paid’) |
|  | Literal | Sie hat Wäsche durch das Haus…  (L) She has thrown laundry around the house. | *geschmissen*  (‘thrown’) | *geschwungen*  (‘swung’) | *gefahren*  (‘driven’) |
| 15 | Idiomatic | Nach Stunden hat er frische Luft…  (L) After hours he has *caught* some fresh air.  (F) After hours he has gasped for air. | *geschnappt*  (‘picked’) | *gefangen*  (‘caught’) | *geatmet*  (‘breathed’) |
|  | Literal | Der Frosch hat gierig nach Fliegen…  (L) The frog has snatched greedily at flies. | *geschnappt*  (‘picked’) | *gehascht*  (‘snatched’) | *geschaut*  (‘looked’) |
| 16 | Idiomatic | Sie hat nach dem Unfall Sterne…  (L) She has *seen* stars after the accident.  (F) She has seen stars after the accident. | *gesehen*  (‘seen’) | *gesichtet*  (‘spotted’) | *gefunden*  (‘found’) |
|  | Literal | Ich habe ihren Bruder nie zuvor…  (L) I have never seen her brother before. | *gesehen*  (‘seen’) | *gefunden*  (‘found’) | *gerettet*  (‘saved’) |
| 17 | Idiomatic | Sie hat immer aufs falsche Pferd…  (L) She has always *sat* on the wrong horse.  (F) She has always bet on the wrong horse. | *gesetzt*  (‘sat’) | *gelehnt*  (‘leaned’) | *gewollt*  (‘wanted’) |
|  | Literal | Sie haben bereits die neuen Pflanzenkeimlinge…  (L) They have already pricked the new seedlings. | *gesetzt*  (‘sat’) | *gesät*  (‘sown’) | *gewollt*  (‘wanted’) |
| 18 | Idiomatic | Er hat immer die zweite Geige…  (L) He has always *played* the second violin.  (F) He has always taken the backseat | *gespielt*  (‘played’) | *gestimmt*  (‘tuned’) | *gehört*  (‘listened’) |
|  | Literal | Sie hat lange in einer Popband…  (L) She has played in a pop band for a long time. | *gespielt*  (‘played’) | *gewirkt*  (‘acted’) | *gedient*  (‘served’) |
| 19 | Idiomatic | Der ehrgeizige Redner hat den Rahmen…  (L) The ambitious speaker has *blown* up the frame.  (F) The ambitious speaker has broken the mold. | *gesprengt*  (‘blown up’) | *geknackt*  (‘cracked’) | *gestaltet*  (‘designed’) |
|  | Literal | Die Bauarbeiter haben die maroden Häuser…  (L) The construction workers have blown up the  shabby houses. | *gesprengt*  (‘blown up’) | *gestürzt*  (‘overthrown’) | *gemeldet*  (‘reported’) |
| 20 | Idiomatic | Sie ist mutig ins kalte Wasser…  (L) She has *jumped* bravely into the cold water.  (F) She has jumped in at the deep end. | *gesprungen*  (‘jumped’) | *gegangen*  (‘gone’) | *gerutscht*  (‘slidden’) |
|  | Literal | Der Passant ist schnell zur Seite…  (L) The pedestrian has quickly jumped aside. | *gesprungen*  (‘jumped’) | *gegangen*  (‘gone’) | *geflohen*  (‘fled’) |
| 21 | Idiomatic | Der kleine Junge hat heimlich Schmiere…  (L) The little boy has secrety *stood* grease.  (F) The little boy has secretly kept watch. | *gestanden*  (‘stood’) | *gehockt*  (‘squatted’) | *getrunken*  (‘drunk’) |
|  | Literal | Ich habe lange in der Schlange…  (L) I have stood in line for a long time. | *gestanden*  (‘stood’) | *gewartet*  (‘waited’) | *gelernt*  (‘studied’) |
| 22 | Idiomatic | Er hat jahrelang nach ihrer Pfeife…  (L) He has *danced* for years after her pipe.  (F) He has danced to her tune for years. | *getanzt*  (‘danced’) | *gesteppt*  (‘tap-danced’) | *gesucht*  (‘searched’) |
|  | Literal | Sie haben stundenlang zu klassischem Swing…  (L) They have danced for hours to classic swing. | *getanzt*  (‘danced’) | *gesungen*  (‘sung’) | *geredet*  (‘talked’) |
| 23 | Idiomatic | Die Psychologin hat genau ins Schwarze…  (L) The psychologist has *hit* exactly into the black.  (F) The psychologist has hit the bull’s eye. | *getroffen*  (‘hit’) | *gezielt*  (‘aimed’) | *gestarrt*  (‘stared’) |
|  | Literal | Er hat Paul beim Kickboxen stark…  (L) He has hit Paul badly during kick boxing. | *getroffen*  (‘hit’) | *gefährdet*  (‘endangered’) | *gefürchtet*  (‘feared’) |
| 24 | Idiomatic | Der Mann hat schnell die Notbremse…  (L) The man has *pulled* the safety brake quickly.  (F) The man has called a halt before it was too late. | *gezogen*  (‘pulled’) | *gelöst*  (‘freed’) | *gesichert*  (‘secured’) |
|  | Literal | Sie haben das Stahlseil noch fester…  (L) They have pulled the steel rope more strongly. | *gezogen*  (‘pulled’) | *gestrafft*  (‘tightened’) | *gesichert*  (‘secured’) |
| **Three-Argument Sentences – Active Voice** | | | | | |
| 25 | Idiomatic | Sie hat ihren Freund zur Kasse ...  (L) She *asked* her boyfriend to come to the cash-register.  (F) She made her boyfriend pay. | *gebeten*  (‘asked’) | *gedrängt*  (‘urged’) | *geholt*  (‘got’) |
|  | Literal | Sie hat ihre Schwester um Hilfe *..*.  (L) She has asked her sister for help. | *gebeten*  (‘asked’) | *gefragt*  (‘asked’) | *gerufen*  (‘called’) |
| 26 | Idiomatic | Er hat sie unter die Haube…  (L) He has *brought* her under the hood.  (F) He has married her off | *gebracht*  (‘brought’) | *geführt*  (‘led’) | *geschafft*  (‘made’) |
|  | Literal | Sie hat den Brief zur Post…  (L) She has brought the letter to the post office. | *gebracht*  (‘brought’) | *geschafft*  (‘made’) | *gepackt*  (‘packed’) |
| 27 | Idiomatic | Er hat ihr kräftig das Genick…  (L) He has *broken* her neck strongly.  (F) He has finished her completely. | gebrochen  (‘broken’) | geteilt  (‘split’) | geküsst  (‘kissed’) |
|  | Literal | Der Aufprall hat ihm den Arm…  (L) The impact has fractured his arm. | *gebrochen*  (‘broken’) | *gestaucht*  (‘jarred’) | *gewirbelt*  (‘spun’) |
| 28 | Idiomatic | Sie hat ihm aus der Hand…  (L) She has *guzzled* out of his hand.  (F) She has eaten out of his hand. | *gefressen*  (‘guzzled’) | *geschlungen*  (‘gorged’) | *gewischt*  (‘wiped’) |
|  | Literal | Die Katze hat Mäuse zum Frühstück…  (L) The cat has guzzled mice for breakfast. | *gefressen*  (‘guzzled’) | *gegessen*  (‘eaten’) | *gezählt*  (‘counted’) |
| 29 | Idiomatic | Sie hat ihm auf den Zahn…  (L) She has *felt* his tooth.  (F) She has sussed him out. | *gefühlt*  (‘felt’) | *gespürt*  (‘sensed’) | *gescheint*  (‘shone’) |
|  | Literal | Sie hat dem Patienten den Puls…  (L) She has checked the patient’s pulse. | *gefühlt*  (‘felt’) | *gespürt*  (‘sensed’) | *geregelt*  (‘regulated’) |
| 30 | Idiomatic | Er hat mächtig Öl ins Feuer…  (L) He has *poured* plenty of oil into the fire.  (F) He added plenty of fuel to the flames. | *gegossen*  (‘poured’) | *gesondert*  (‘assorted’) | *gefüllt*  (‘filled’) |
|  | Literal | Sie hat der Nachbarin die Blumen…  (L) She has watered the neighbor’s flowers. | *gegossen*  (‘poured’) | *gewässert*  (‘watered’) | *gepflegt*  (‘fostered’) |
| 31 | Idiomatic | Sie hat ihm in die Karten…  (L) She has *peeked* into his cards.  (F) She has looked at his cards. | *geguckt*  (‘peeked’) | *gespäht*  (‘spied’) | *gemalt*  (‘painted’) |
|  | Literal | Sie hat für ihn nach Autos…  (L) She looked out for cars for him. | *geguckt*  (‘peeked’) | *gespäht*  (‘spied’) | *gehorcht*  (‘listened’) |
| 32 | Idiomatic | Er hat es an den Nagel…  (L) He has *hung* it on the nail.  (F) He has given it up. | *gehängt*  (‘hung’) | *gebaumelt*  (‘dangled’) | *gesteckt*  (‘plugged’) |
|  | Literal | Ich habe die Hemden auf die Kleiderbügel…  (L) I have hung the shirts on hangers. | *gehängt*  (‘hung’) | *gebaumelt*  (‘dangled’) | *gesteckt*  (‘plugged’) |
| 33 | Idiomatic | Er hat sie in den Himmel…  (L) He has *lifted* her up into the sky.  (F) He has set her on a pedestal. | *gehoben*  (‘lifted’) | *gewuchtet*  (‘balanced’) | *geflogen*  (‘flown’) |
|  | Literal | Er hat ihr den schweren Karton…  (L) He has carried the heavy box for her. | *gehoben*  (‘lifted’) | *geschleppt*  (‘dragged’) | *geschaukelt*  (‘swung’) |
| 34 | Idiomatic | Er hat die Katze im Sack…  (L) He has *bought* the cat in the sack.  (F) He has bought a pig in a poke. | *gekauft*  (‘bought’) | *gehandelt*  (‘traded’) | *getragen*  (‘carried’) |
|  | Literal | Sie hat ihm wieder neue Klamotten…  (L) She has bought him new clothes again. | *gekauft*  (‘bought’) | *gehandelt*  (‘traded’) | *gestrickt*  (‘knitted’) |
| 35 | Idiomatic | Sie hat ihm auf die Finger…  (L) She has *knocked* on his fingers.  (F) She has rapped his knuckles. | *geklopft*  (‘knocked’) | *gehämmert*  (‘hammered’) | *geleuchtet*  (‘glowed’) |
|  | Literal | Er hat ihr an die Tür…  (L) He has knocked on her door. | *geklopft*  (‘knocked’) | *gepocht*  (‘throbbed’) | *gepinselt*  (‘brushed’) |
| 36 | Idiomatic | Er hat ihr nie ein Haar…  (L) He has never *bent* her a hair.  (F) He has never laid a finger on her. | *gekrümmt*  (‘bent’) | *gebogen*  (‘curved’) | *gerupft*  (‘plucked’) |
|  | Literal | Die Natur hat uns die Banane…  (L) Nature has bent us the banana. | *gekrümmt*  (‘bent’) | *gebogen*  (‘curved’) | *gezeugt*  (‘fathered’) |
| 37 | Idiomatic | Er hat die Angestellte aufs Eis…  (L) He has *laid* the employee on ice.  (F) He has put the employee on the back burner. | *gelegt*  (‘laid’) | *gestellt*  (‘placed’) | *gewählt*  (‘selected’) |
|  | Literal | Er hat den Arm um sie…  (L) He has put the arm around her. | *gelegt*  (‘laid’) | *gemacht*  (‘made’) | *geworfen*  (‘thrown’) |
| 38 | Idiomatic | Sie hat der Freundin die Augen…  (L) She has *opened* the friend’s eyes.  (F) She has given the friend a reality check. | *geöffnet*  (‘opened’) | *geklärt*  (‘cleared’) | *gebessert*  (‘improved’) |
|  | Literal | Er hat ihr vorsichtig die Schmuckschatulle…  (L) He has opened her jewelry case carefully. | *geöffnet*  (‘opened’) | *geklappt*  (‘worked out’) | *geschätzt*  (‘appreciated’) |
| 39 | Idiomatic | Er hat ihr nicht das Wasser…  (L) He has not *handed* her the water.  (F) He was nowhere near on a par with her. | *gereicht*  (‘handed’) | *gegeben*  (‘given’) | *geprüft*  (‘checked’) |
|  | Literal | Sie hat dem Mann den Brotkorb…  (L) She has handed the breadbasket to the man. | *gereicht*  (‘handed’) | *gegeben*  (‘given’) | *gestattet*  (‘allowed’) |
| 40 | Idiomatic | Er hat sie aus der Bahn…  (L) He has *shot* her out of the lane.  (F) He has knocked her off her perch. | *geschleudert*  (‘shot’) | *geschmettert*  (‘blared’) | *gelenkt*  (‘steered’) |
|  | Literal | Sie hat Konfetti in die Luft…  (L) She has shot confetti into the air. | *geschleudert*  (‘shot’) | *geschmettert*  (‘blared’) | *gestapelt*  (‘stacked’) |
| 41 | Idiomatic | Sie hat ihn sehr ins Herz…  (L) She *shut* him badly into the heart.  (F) She badly took him to her heart. | *geschlossen*  (‘shut’) | *gesperrt*  (‘locked’) | *gezeichnet*  (‘drawn’) |
|  | Literal | Er hat der Frau das Fenster…  (L) He has shut the woman’s window. | *geschlossen*  (‘shut’) | *gekippt*  (‘canted’) | *gebaut*  (‘built’) |
| 42 | Idiomatic | Er hat die Frau aufs Abstellgleis…  (L) He has *scooped* the woman onto the siding.  (F) He has put the woman out to grass. | *geschoben*  (‘scooped’) | *gerückt*  (‘moved’) | *gebunden*  (‘bound’) |
|  | Literal | Er hat der Oma den Wagen…  (L) He has scooped the grandmother’s cart. | *geschoben*  (‘scooped’) | *gerollt*  (‘rolled’) | *geordnet*  (‘arranged’) |
| 43 | Idiomatic | Er hat sie auf die Folter…  (L) He has *strained* her on the torture.  (F) He has kept her on tenterhooks. | *gespannt*  (‘strained’) | *gedehnt*  (‘stretched’) | *gefesselt*  (‘constrained’) |
|  | Literal | Er hat Pferde an den Wagen…  (L) He has strained horses onto the wagon. | *gespannt*  (‘strained’) | *gekettet*  (‘chained’) | *gewöhnt*  (‘accustomed’) |
| 44 | Idiomatic | Er ist der Geliebten aufs Dach…  (L) He has *gone* up onto the lover’s roof.  (F) He has hauled the lover over the coals. | *gestiegen*  (‘gone up’) | *geklettert*  (‘climbed’) | *gefolgt*  (‘followed’) |
|  | Literal | Er ist Pferden auf den Rücken…  (L) He has gone up on horses’ backs. | *gestiegen*  (‘gone up’) | *gekrabbelt*  (‘crawled’) | *gefallen*  (‘fallen’) |
| 45 | Idiomatic | Er hat sie vor den Kopf…  (L) He has *pushed* her before the head.  (F) He has kicked her in the teeth. | *gestoßen*  (‘pushed’) | *gerammt*  (‘rammed’) | *gesenkt*  (‘sunk’) |
|  | Literal | Er hat den Dolch ins Herz…  (L) He has thrust the dagger in the heart. | *gestoßen*  (‘pushed’) | *gebohrt*  (‘drilled’) | *gefügt*  (‘jointed’) |
| 46 | Idiomatic | Sie hat dem Jungen die Flügel…  (L) She has *cropped* the boy’s wings.  (F) She has clipped the boy’s wings. | *gestutzt*  (‘cropped’) | *gekürzt*  (‘shortened’) | *gekämmt*  (‘combed’) |
|  | Literal | Sie hat ihm morgens den Bart…  (L) She has cut back his beard in the mornings. | *gestutzt*  (‘cropped’) | *getrimmt*  (‘trimmed’) | *gefärbt*  (‘colored’) |
| 47 | Idiomatic | Er ist ihr auf die Zehen…  (L) He has *stepped* on her toes.  (F) He has trod on her toes. | *getreten*  (‘stepped’) | *getrampelt*  (‘clumped’) | *gerast*  (‘rushed’) |
|  | Literal | Sie ist ihm in die Hacken…  (L) She has stepped in his heel. | *getreten*  (‘stepped’) | *gestampft*  (‘stamped’) | *gehüpft*  (‘jumped’) |
| 48 | Idiomatic | Er hat sie in die Enge…  (L) He has *chased* her into the narrow.  (F) He has driven her into a corner. | *getrieben*  (‘chased’) | *gehetzt*  (‘rushed’) | *gekämpft*  (‘fought’) |
|  | Literal | Der Hund hat die Schafe heimwärts…  (L) The dog has chased the sheep homeward. | *getrieben*  (‘chased’) | *geleitet*  (‘led’) | *gelockt*  (‘lured’) |
| 49 | Idiomatic | Sie hat ihm gründlich den Kopf…  (L) She thoroughly *washed* his head.  (F) She has given him a piece of her mind. | *gewaschen*  (‘washed’) | *gereinigt*  (‘cleaned’) | *gemessen*  (‘measured’) |
|  | Literal | Er hat der Tochter die Haare…  (L) He has washed the daughter’s hair. | *gewaschen*  (‘washed’) | *gesäubert*  (‘cleaned’) | *gebürstet*  (‘brushed’) |
| 50 | Idiomatic | Sie hat ihn in die Schranken…  (L) She has *shown* him the barriers.  (F) She has put him in his place. | *gewiesen*  (‘shown’) | *geschickt*  (‘sent’) | *geneigt*  (‘leaned’) |
|  | Literal | Er hat ihr wütend die Tür…  (L) He has shown her the door angrily. | *gewiesen*  (‘shown’) | *geschildert*  (‘depicted’) | *geliefert*  (‘delivered’) |
| 51 | Idiomatic | Die Frau hat ihm die Krallen…  (L) The woman has *shown* him the claws.  (F) The woman has bared him her teeth. | *gezeigt*  (‘shown’) | *gedeutet*  (‘interpreted’) | *genehmigt*  (‘approved’) |
|  | Literal | Sie hat dem Touristen den Weg…  (L) She has shown the tourist the path. | *gezeigt*  (‘shown’) | *gedeutet*  (‘interpreted’) | *geflutet*  (‘flooded’) |
| 52 | Idiomatic | Sie hat ihn in die Knie…  (L) She has *forced* him on the knees.  (F) She has brought him to his knees. | *gezwungen*  (‘forced’) | *geboten*  (‘demanded’) | *gebeugt*  (‘bent’) |
|  | Literal | Er hat das Mädchen zur Prositution…  (L) He has forced the girl into prostitution. | *gezwungen*  (‘forced’) | *genötigt*  (‘coerced’) | *geladen*  (‘charged’) |
| **Two-Argument Sentences – Passive Voice** | | | | | |
| 1 | Idiomatic | In den sauren Apfel wurde von ihm… | *gebissen* | *gespuckt* | *geschnitten* |
|  | Literal | Der Postbote wurde von dem Hund bereits… | *gebissen* | *gezwickt* | *gerochen* |
| 2 | Idiomatic | Ins gleiche Horn wurde von ihnen beiden… | *geblasen* | *gehaucht* | *geseufzt* |
|  | Literal | Das Saxophon wurde von dem talentierten Musiker… | *geblasen* | *gepustet* | *geputzt* |
| 3 | Idiomatic | Die Schulbank wurde gemeinsam von den Jungs… | *gedrückt* | *gepresst* | *genutzt* |
|  | Literal | Alle ihre Pickel wurden von ihr wund… | *gedrückt* | *gekratzt* | *gelegen* |
| 4 | Idiomatic | Nach den Sternen wurde immer von ihr … | *gegriffen* | *gelangt* | *gelebt* |
|  | Literal | Nach den Bonbons wurde von dem Mädchen… | *gegriffen* | *gelangt* | *getastet* |
| 5 | Idiomatic | Der Schnabel wurde schnell von dem Jungen… | *gehalten* | *gestützt* | *geschüttelt* |
|  | Literal | Die Hundeleine wurde von mir sehr kurz … | *gehalten* | *gefasst* | *gebraucht* |
| 6 | Idiomatic | An der Strippe wurde lange von ihm… | *gehangen* | *gependelt* | *gesprochen* |
|  | Literal | Am Haken wurde lange von dem Regenschirm… | *gehangen* | *gependelt* | *gewackelt* |
| 7 | Idiomatic | Auf den Putz wurde ordentlich von ihm… | *gehauen* | *gehackt* | *gestrichen* |
|  | Literal | Auf den Tisch wurde kräftig von ihm… | *gehauen* | *getrommelt* | *gemurmelt* |
| 8 | Idiomatic | Nach Blut wurde von dem jungen Mädchen… | *gelechzt* | *gegiert* | *geschielt* |
|  | Literal | Nach Wasser wurde von dem durstigen Marathonläufer… | *gelechzt* | *gegiert* | *gewühlt* |
| 9 | Idiomatic | Der Schleier wurde endlich von der Frau… | *gelüftet* | *gerafft* | *gestreift* |
|  | Literal | Das miefende Zimmer wurde nie von ihr… | *gelüftet* | *getrocknet* | *gemietet* |
| 10 | Idiomatic | Der Mund wurde von ihr zu voll… | *genommen* | *getan* | *gelassen* |
|  | Literal | Die Tabletten wurden von der abhängigen Frau… | *genommen* | *gefordert* | *gelassen* |
| 11 | Idiomatic | Das Feld wurde nach Tagen von ihr… | *geräumt* | *geleert* | *gekehrt* |
|  | Literal | Das Zimmer wurde letztendlich von dem Mädchen… | *geräumt* | *gewechselt* | *gemeint* |
| 12 | Idiomatic | Das kleine Kind wurde mächtig vom Teufel… | *geritten* | *gejagt* | *geärgert* |
|  | Literal | Das junge Pferd wurde täglich von ihr… | *geritten* | *gezügelt* | *gemocht* |
| 13 | Idiomatic | Mächtig Schaum wurde von der jungen Frau… | *geschlagen* | *gerpügelt* | *gebildet* |
|  | Literal | Er wurde von ihr aus purer Notwehr… | *geschlagen* | *geohrfeigt* | *geschützt* |
| 14 | Idiomatic | Der Laden wurde ganz alleine von ihm… | *geschmissen* | *geschwungen* | *gezahlt* |
|  | Literal | Wäsche wurde von ihr durch das Haus… | *geschmissen* | *geschwungen* | *gefahren* |
| 15 | Idiomatic | Frische Luft wurde nach Stunden von ihm… | *geschnappt* | *gefangen* | *geatmet* |
|  | Literal | Nach Fliegen wurde gierig von dem Frosch… | *geschnappt* | *gehascht* | *geschaut* |
| 16 | Idiomatic | Sterne wurden von ihr nach dem Unfall… | *gesehen* | *gesichtet* | *gefunden* |
|  | Literal | Ihr Bruder wurde von mir nie zuvor… | *gesehen* | *gefunden* | *gerettet* |
| 17 | Idiomatic | Aufs falsche Pferd wurde immer von ihr … | *gesetzt* | *gelehnt* | *gewollt* |
|  | Literal | Die neuen Pflanzenkeime wurden bereits von ihnen… | *gesetzt* | *gesät* | *gewollt* |
| 18 | Idiomatic | Die zweite Geige wurde immer von ihm… | *gespielt* | *gestimmt* | *gehört* |
|  | Literal | In einer Popband wurde lange von ihr… | *gespielt* | *gewirkt* | *gedient* |
| 19 | Idiomatic | Der Rahmen wurde von dem ehrgeizigen Redner… | *gesprengt* | *geknackt* | *gestaltet* |
|  | Literal | Die maroden Häuser wurden von den Bauarbeitern… | *gesprengt* | *gestürzt* | *gemeldet* |
| 20 | Idiomatic | Ins kalte Wasser wurde mutig von ihr… | *gesprungen* | *gegangen* | *gerutscht* |
|  | Literal | Zur Seite wurde schnell von dem Passanten… | *gesprungen* | *gegangen* | *geflohen* |
| 21 | Idiomatic | Schmiere wurde heimlich von dem kleinen Jungen… | *gestanden* | *gehockt* | *getrunken* |
|  | Literal | In der Schlange wurde lange von mir… | *gestanden* | *gewartet* | *gelernt* |
| 22 | Idiomatic | Nach ihrer Pfeife wurde jahrelang von ihm… | *getanzt* | *gesteppt* | *gesucht* |
|  | Literal | Zu klassischem Swing wurde stundenlang von ihnen… | *getanzt* | *gesungen* | *geredet* |
| 23 | Idiomatic | Ins Schwarze wurde genau von der Psychologin… | *getroffen* | *gezielt* | *gestarrt* |
|  | Literal | Paul wurde von ihm beim Kickboxen stark… | *getroffen* | *gefährdet* | *gefürchtet* |
| 24 | Idiomatic | Die Notbremse wurde schnell von dem Mann… | *gezogen* | *gelöst* | *gesichert* |
|  | Literal | Das Stahlseil wurde noch fester von ihnen… | *gezogen* | *gestrafft* | *gesichert* |
| **Three-Argument Sentences – Passive Voice** | | | | | |
| 25 | Idiomatic | Ihr Freund wurde von ihr zur Kasse... | *gebeten* | *gedrängt* | *geholt* |
|  | Literal | Ihre Schwester wurde von ihr um Hilfe*..*. | *gebeten* | *gefragt* | *gerufen* |
| 26 | Idiomatic | Sie wurde von ihm unter die Haube… | *gebracht* | *geführt* | *geschafft* |
|  | Literal | Der Brief wurde von ihr zur Post … | *gebracht* | *geschafft* | *gepackt* |
| 27 | Idiomatic | Ihr wurde von ihm kräftig das Genick … | *gebrochen* | *geteilt* | *geküsst* |
|  | Literal | Der Arm wurde ihm von dem Aufprall … | *gebrochen* | *gestaucht* | *gewirbelt* |
| 28 | Idiomatic | Ihm wurde von ihr aus der Hand … | *gefressen* | *geschlungen* | *gewischt* |
|  | Literal | Mäuse wurden von der Katze zum Frühstück… | *gefressen* | *gegessen* | *gezählt* |
| 29 | Idiomatic | Ihm wurde von ihr auf den Zahn… | *gefühlt* | *gespürt* | *gescheint* |
|  | Literal | Dem Patienten wurde von ihr der Puls… | *gefühlt* | *gespürt* | *geregelt* |
| 30 | Idiomatic | Mächtig Öl wurde von ihm ins Feuer … | *gegossen* | *gesondert* | *gefüllt* |
|  | Literal | Der Nachbarin wurden von ihr die Blumen… | *gegossen* | *gewässert* | *gepflegt* |
| 31 | Idiomatic | Ihm wurde von ihr in die Karten… | *geguckt* | *gespäht* | *gemalt* |
|  | Literal | Für ihn wurde von ihr nach Autos… | *geguckt* | *gespäht* | *gehorcht* |
| 32 | Idiomatic | Es wurde von ihm an den Nagel… | *gehängt* | *gebaumelt* | *gesteckt* |
|  | Literal | Die Hemden wurden von mir auf Kleiderbügel… | *gehängt* | *gebaumelt* | *gesteckt* |
| 33 | Idiomatic | Sie wurde von ihm in den Himmel… | *gehoben* | *gewuchtet* | *geflogen* |
|  | Literal | Ihr wurde von ihm der schwere Karton… | *gehoben* | *geschleppt* | *geschaukelt* |
| 34 | Idiomatic | Die Katze wurde von ihm im Sack… | *gekauft* | *gehandelt* | *getragen* |
|  | Literal | Ihm wurden von ihr wieder neue Klamotten… | *gekauft* | *gehandelt* | *gestrickt* |
| 35 | Idiomatic | Ihm wurde von ihr auf die Finger… | *geklopft* | *gehämmert* | *geleuchtet* |
|  | Literal | Ihr wurde von ihm an die Tür… | *geklopft* | *gepocht* | *gepinselt* |
| 36 | Idiomatic | Ihr wurde von ihm nie ein Haar… | *gekrümmt* | *gebogen* | *gerupft* |
|  | Literal | Uns wurde von der Natur die Banane… | *gekrümmt* | *gebogen* | *gezeugt* |
| 37 | Idiomatic | Die Angestellte wurde von ihm aufs Eis… | *gelegt* | *gestellt* | *gewählt* |
|  | Literal | Der Arm wurde von ihm um sie… | *gelegt* | *gemacht* | *geworfen* |
| 38 | Idiomatic | Der Freundin wurden von ihr die Augen… | *geöffnet* | *geklärt* | *gebessert* |
|  | Literal | Ihr wurde von ihm vorsichtig die Schmuckschatulle… | *geöffnet* | *geklappt* | *geschätzt* |
| 39 | Idiomatic | Ihr wurde von ihm nicht das Wasser… | *gereicht* | *gegeben* | *geprüft* |
|  | Literal | Dem Mann wurde von ihr der Brotkorb… | *gereicht* | *gegeben* | *gestattet* |
| 40 | Idiomatic | Sie wurde von ihm aus der Bahn… | *geschleudert* | *geschmettert* | *gelenkt* |
|  | Literal | Konfetti wurde von ihr in die Luft… | *geschleudert* | *geschmettert* | *gestapelt* |
| 41 | Idiomatic | Er wurde von ihr sehr ins Herz… | *geschlossen* | *gesperrt* | *gezeichnet* |
|  | Literal | Der Frau wurde von ihm das Fenster… | *geschlossen* | *gekippt* | *gebaut* |
| 42 | Idiomatic | Die Frau wurde von ihm aufs Abstellgleis… | *geschoben* | *gerückt* | *gebunden* |
|  | Literal | Der Oma wurde von ihm der Wagen… | *geschoben* | *gerollt* | *geordnet* |
| 43 | Idiomatic | Sie wurde von ihm auf die Folter… | *gespannt* | *gedehnt* | *gefesselt* |
|  | Literal | Pferde wurden von ihm an den Wagen… | *gespannt* | *gekettet* | *gewöhnt* |
| 44 | Idiomatic | Der Geliebten wurde von ihm aufs Dach… | *gestiegen* | *geklettert* | *gefolgt* |
|  | Literal | Pferden wurde von ihm auf den Rücken… | *gestiegen* | *gekrabbelt* | *gefallen* |
| 45 | Idiomatic | Er wurde von ihr vor den Kopf… | *gestoßen* | *gerammt* | *gesenkt* |
|  | Literal | Der Dolch wurde von ihm ins Herz… | *gestoßen* | *gebohrt* | *gefügt* |
| 46 | Idiomatic | Dem Jungen wurden von ihr die Flügel… | *gestutzt* | *gekürzt* | *gekämmt* |
|  | Literal | Ihm wurde von ihr morgens der Bart… | *gestutzt* | *getrimmt* | *gefärbt* |
| 47 | Idiomatic | Ihr wurde von ihm auf die Zehen… | *getreten* | *getrampelt* | *gerast* |
|  | Literal | Ihm wurde von ihr in die Hacken… | *getreten* | *gestampft* | *gehüpft* |
| 48 | Idiomatic | Sie wurde von ihm in die Enge… | *getrieben* | *gehetzt* | *gekämpft* |
|  | Literal | Die Schafe wurden von dem Hund heimwärts… | *getrieben* | *geleitet* | *gelockt* |
| 49 | Idiomatic | Ihm wurde von ihr gründlich der Kopf… | *gewaschen* | *gereinigt* | *gemessen* |
|  | Literal | Der Tochter wurden von ihm die Haare… | *gewaschen* | *gesäubert* | *gebürstet* |
| 50 | Idiomatic | Er wurde von ihr in die Schranken… | *gewiesen* | *geschickt* | *geneigt* |
|  | Literal | Ihr wurde von ihm wütend die Tür… | *gewiesen* | *geschildert* | *geliefert* |
| 51 | Idiomatic | Ihm wurden von der Frau die Krallen… | *gezeigt* | *gedeutet* | *genehmigt* |
|  | Literal | Dem Touristen wurde von ihr der Weg… | *gezeigt* | *gedeutet* | *geflutet* |
| 52 | Idiomatic | Er wurde von ihr in die Knie… | *gezwungen* | *geboten* | *gebeugt* |
|  | Literal | Das Mädchen wurde von ihm zur Prostitution… | *gezwungen* | *genötigt* | *geladen* |

**Appendix B**

*Fixed Effects of the Predictors in the Linear Mixed-Effect Model for Log-Transformed Reaction Times in Experiment 1.*

|  | **Estimate** | **Std. Error** | **df** | ***t-*value** | ***p*** |
| --- | --- | --- | --- | --- | --- |
| Intercept: literal, active, three, 6 letters | 6.805 | 0.15 | 115 | 46.71 | < .0001 |
| Sentence Type (idiomatic) | -0.110 | 0.04 | 100 | -2.83 | .0057 |
| Voice (passive) | 0.057 | 0.02 | 2945 | 3.47 | .0005 |
| Arguments (two) | -0.005 | 0.04 | 120 | -0.12 | .9052 |
| Target Length (per additional letter) | 0.037 | 0.02 | 100 | 2.43 | .0271 |
| Voice (passive) × Arguments (two) | 0.121 | 0.02 | 2945 | 5.00 | < .0001 |

*Note.* The intercept refers to literal sentences in active voice with three arguments and targets with 6 letters.

**Appendix C**

*Experimental sentences in active and passive voice of Experiment 2*

| **Two-Argument Sentences – Active Voice** | | | | | |
| --- | --- | --- | --- | --- | --- |
| **Sentence Pair** | **Sentence Type** | **Sentence** | **Target Verb** | | |
|  |  |  | **Figurative** | **Semantic** | **Unrelated** |
| 1 | Idiomatic | In den sauren Apfel hat er… | *gebissen* | *gespuckt* | *geschnitten* |
|  | Literal | Den Postboten hat der Hund bereits… | *gebissen* | *gezwickt* | *gerochen* |
| 2 | Idiomatic | Ins gleiche Horn haben sie beide… | *geblasen* | *gehaucht* | *geseufzt* |
|  | Literal | Das Saxophon hat der talentierte Musiker… | *geblasen* | *gepustet* | *geputzt* |
| 3 | Idiomatic | Die Schulbank haben die Jungs gemeinsam… | *gedrückt* | *gepresst* | *genutzt* |
|  | Literal | Alle ihre Pickel hat sie wund… | *gedrückt* | *gekratzt* | *gelegen* |
| 4 | Idiomatic | Nach den Sternen hat sie immer… | *gegriffen* | *gelangt* | *gelebt* |
|  | Literal | Nach den Bonbons hat das Mädchen… | *gegriffen* | *gelangt* | *getastet* |
| 5 | Idiomatic | Den Schnabel hat der Junge schnell… | *gehalten* | *gestützt* | *geschüttelt* |
|  | Literal | Die Hundeleine habe ich sehr kurz … | *gehalten* | *gefasst* | *gebraucht* |
| 6 | Idiomatic | An der Strippe hat er lange… | *gehangen* | *gependelt* | *gesprochen* |
|  | Literal | Am Haken hat der Regenschirm lange… | *gehangen* | *gependelt* | *gewackelt* |
| 7 | Idiomatic | Auf den Putz hat er ordentlich… | *gehauen* | *gehackt* | *gestrichen* |
|  | Literal | Auf den Tisch hat er kräftig … | *gehauen* | *getrommelt* | *gemurmelt* |
| 8 | Idiomatic | Nach Blut hat das junge Mädchen… | *gelechzt* | *gegiert* | *geschielt* |
|  | Literal | Nach Wasser hat der durstige Marathonläufer… | *gelechzt* | *gegiert* | *gewühlt* |
| 9 | Idiomatic | Den Schleier hat die Frau endlich… | *gelüftet* | *gerafft* | *gestreift* |
|  | Literal | Das miefende Zimmer hat sie nie… | *gelüftet* | *getrocknet* | *gemietet* |
| 10 | Idiomatic | Den Mund hat sie zu voll… | *genommen* | *getan* | *gelassen* |
|  | Literal | Die Tabletten hat die abhängige Frau… | *genommen* | *gefordert* | *gelassen* |
| 11 | Idiomatic | Das Feld hat sie nach Tagen … | *geräumt* | *geleert* | *gekehrt* |
|  | Literal | Das Zimmer hat das Mädchen letztendlich… | *geräumt* | *gewechselt* | *gemeint* |
| 12 | Idiomatic | Das kleine Kind hat der Teufel… | *geritten* | *gejagt* | *geärgert* |
|  | Literal | Das junge Pferd hat sie täglich … | *geritten* | *gezügelt* | *gemocht* |
| 13 | Idiomatic | Mächtig Schaum hat die junge Frau… | *geschlagen* | *gerpügelt* | *gebildet* |
|  | Literal | Ihn hat sie aus purer Notwehr… | *geschlagen* | *geohrfeigt* | *geschützt* |
| 14 | Idiomatic | Den Laden hat er ganz alleine … | *geschmissen* | *geschwungen* | *gezahlt* |
|  | Literal | Wäsche hat sie durch das Haus… | *geschmissen* | *geschwungen* | *gefahren* |
| 15 | Idiomatic | Frische Luft hat sie nach Stunden … | *geschnappt* | *gefangen* | *geatmet* |
|  | Literal | Nach Fliegen hat der Frosch gierig … | *geschnappt* | *gehascht* | *geschaut* |
| 16 | Idiomatic | Sterne hat sie nach dem Unfall… | *gesehen* | *gesichtet* | *gefunden* |
|  | Literal | Ihren Bruder habe ich nie zuvor… | *gesehen* | *gefunden* | *gerettet* |
| 17 | Idiomatic | Aufs falsche Pferd hat sie immer … | *gesetzt* | *gelehnt* | *gewollt* |
|  | Literal | Die neuen Pflanzenkeime haben sie bereits … | *gesetzt* | *gesät* | *gewollt* |
| 18 | Idiomatic | Die zweite Geige hat er immer … | *gespielt* | *gestimmt* | *gehört* |
|  | Literal | In einer Popband hat sie lange … | *gespielt* | *gewirkt* | *gedient* |
| 19 | Idiomatic | Den Rahmen hat der ehrgeizigen Redner… | *gesprengt* | *geknackt* | *gestaltet* |
|  | Literal | Die maroden Häuser haben die Bauarbeiter… | *gesprengt* | *gestürzt* | *gemeldet* |
| 20 | Idiomatic | Ins kalte Wasser ist sie mutig … | *gesprungen* | *gegangen* | *gerutscht* |
|  | Literal | Zur Seite ist der Passant schnell … | *gesprungen* | *gegangen* | *geflohen* |
| 21 | Idiomatic | Schmiere hat der kleine Junge heimlich… | *gestanden* | *gehockt* | *getrunken* |
|  | Literal | In der Schlange habe ich lange … | *gestanden* | *gewartet* | *gelernt* |
| 22 | Idiomatic | Nach ihrer Pfeife hat er jahrelang … | *getanzt* | *gesteppt* | *gesucht* |
|  | Literal | Zu klassischem Swing haben sie stundenlang … | *getanzt* | *gesungen* | *geredet* |
| 23 | Idiomatic | Ins Schwarze hat die Psychologin genau… | *getroffen* | *gezielt* | *gestarrt* |
|  | Literal | Paul hat er beim Kickboxen stark… | *getroffen* | *gefährdet* | *gefürchtet* |
| 24 | Idiomatic | Die Notbremse hat der Mann schnell… | *gezogen* | *gelöst* | *gesichert* |
|  | Literal | Das Stahlseil haben sie noch fester … | *gezogen* | *gestrafft* | *gesichert* |
| **Three-Argument Sentences – Active Voice** | | | | | |
| 25 | Idiomatic | Zur Kasse hat sie ihren Freund... | *gebeten* | *gedrängt* | *geholt* |
|  | Literal | Um Hilfe hat sie ihre Schwester... | *gebeten* | *gefragt* | *gerufen* |
| 26 | Idiomatic | Unter die Haube hat er sie … | *gebracht* | *geführt* | *geschafft* |
|  | Literal | Zur Post hat sie den Brief … | *gebracht* | *geschafft* | *gepackt* |
| 27 | Idiomatic | Das Genick hat er ihr kräftig … | *gebrochen* | *geteilt* | *geküsst* |
|  | Literal | Den Arm hat der Aufprall ihm… | *gebrochen* | *gestaucht* | *gewirbelt* |
| 28 | Idiomatic | Aus der Hand hat sie ihm … | *gefressen* | *geschlungen* | *gewischt* |
|  | Literal | Zum Frühstück hat die Katze Mäuse… | *gefressen* | *gegessen* | *gezählt* |
| 29 | Idiomatic | Auf den Zahn hat sie ihm… | *gefühlt* | *gespürt* | *gescheint* |
|  | Literal | Den Puls hat sie dem Patienten… | *gefühlt* | *gespürt* | *geregelt* |
| 30 | Idiomatic | Ins Feuer hat er mächtig Öl … | *gegossen* | *gesondert* | *gefüllt* |
|  | Literal | Die Blumen hat sie der Nachbarin… | *gegossen* | *gewässert* | *gepflegt* |
| 31 | Idiomatic | In die Karten hat sie ihm… | *geguckt* | *gespäht* | *gemalt* |
|  | Literal | Nach Autos hat sie für ihn… | *geguckt* | *gespäht* | *gehorcht* |
| 32 | Idiomatic | An den Nagel hat er es… | *gehängt* | *gebaumelt* | *gesteckt* |
|  | Literal | Auf Kleiderbügel habe ich die Hemden… | *gehängt* | *gebaumelt* | *gesteckt* |
| 33 | Idiomatic | In den Himmel hat er sie… | *gehoben* | *gewuchtet* | *geflogen* |
|  | Literal | Den schweren Karton hat er ihr… | *gehoben* | *geschleppt* | *geschaukelt* |
| 34 | Idiomatic | Im Sack hat er die Katze… | *gekauft* | *gehandelt* | *getragen* |
|  | Literal | Neue Klamotten hat sie ihm wieder… | *gekauft* | *gehandelt* | *gestrickt* |
| 35 | Idiomatic | Auf die Finger hat sie ihm… | *geklopft* | *gehämmert* | *geleuchtet* |
|  | Literal | An die Tür hat er ihr… | *geklopft* | *gepocht* | *gepinselt* |
| 36 | Idiomatic | Ein Haar hat er ihr nie… | *gekrümmt* | *gebogen* | *gerupft* |
|  | Literal | Die Banane hat die Natur uns… | *gekrümmt* | *gebogen* | *gezeugt* |
| 37 | Idiomatic | Aufs Eis hat er die Angestellte… | *gelegt* | *gestellt* | *gewählt* |
|  | Literal | Um sie hat er den Arm… | *gelegt* | *gemacht* | *geworfen* |
| 38 | Idiomatic | Die Augen hat sie der Freundin… | *geöffnet* | *geklärt* | *gebessert* |
|  | Literal | Die Schmuckschatulle hat er ihr vorsichtig… | *geöffnet* | *geklappt* | *geschätzt* |
| 39 | Idiomatic | Das Wasser hat er ihr nicht… | *gereicht* | *gegeben* | *geprüft* |
|  | Literal | Den Brotkorb hat sie dem Mann… | *gereicht* | *gegeben* | *gestattet* |
| 40 | Idiomatic | Aus der Bahn hat er sie… | *geschleudert* | *geschmettert* | *gelenkt* |
|  | Literal | In die Luft hat sie Konfetti… | *geschleudert* | *geschmettert* | *gestapelt* |
| 41 | Idiomatic | Ins Herz hat sie ihn sehr… | *geschlossen* | *gesperrt* | *gezeichnet* |
|  | Literal | Das Fenster hat er der Frau… | *geschlossen* | *gekippt* | *gebaut* |
| 42 | Idiomatic | Aufs Abstellgleis hat er die Frau… | *geschoben* | *gerückt* | *gebunden* |
|  | Literal | Den Wagen hat er der Oma… | *geschoben* | *gerollt* | *geordnet* |
| 43 | Idiomatic | Auf die Folter hat er sie… | *gespannt* | *gedehnt* | *gefesselt* |
|  | Literal | An den Wagen hat er die Pferde… | *gespannt* | *gekettet* | *gewöhnt* |
| 44 | Idiomatic | Aufs Dach ist er der Geliebten… | *gestiegen* | *geklettert* | *gefolgt* |
|  | Literal | Auf den Rücken ist er Pferden… | *gestiegen* | *gekrabbelt* | *gefallen* |
| 45 | Idiomatic | Vor den Kopf hat er sie… | *gestoßen* | *gerammt* | *gesenkt* |
|  | Literal | Ins Herz hat er den Dolch… | *gestoßen* | *gebohrt* | *gefügt* |
| 46 | Idiomatic | Die Flügel hat sie dem Jungen… | *gestutzt* | *gekürzt* | *gekämmt* |
|  | Literal | Den Bart hat sie ihm morgens… | *gestutzt* | *getrimmt* | *gefärbt* |
| 47 | Idiomatic | Auf die Zehen ist er ihr… | *getreten* | *getrampelt* | *gerast* |
|  | Literal | In die Hacken ist sie ihm… | *getreten* | *gestampft* | *gehüpft* |
| 48 | Idiomatic | In die Enge hat er sie… | *getrieben* | *gehetzt* | *gekämpft* |
|  | Literal | Heimwärts hat der Hund die Schafe… | *getrieben* | *geleitet* | *gelockt* |
| 49 | Idiomatic | Den Kopf hat sie ihm gründlich… | *gewaschen* | *gereinigt* | *gemessen* |
|  | Literal | Die Haare hat er der Tochter… | *gewaschen* | *gesäubert* | *gebürstet* |
| 50 | Idiomatic | In die Schranken hat sie ihn… | *gewiesen* | *geschickt* | *geneigt* |
|  | Literal | Die Tür hat er ihr wütend… | *gewiesen* | *geschildert* | *geliefert* |
| 51 | Idiomatic | Die Krallen hat die Frau ihm… | *gezeigt* | *gedeutet* | *genehmigt* |
|  | Literal | Den Weg hat sie dem Touristen… | *gezeigt* | *gedeutet* | *geflutet* |
| 52 | Idiomatic | In die Knie hat sie ihn… | *gezwungen* | *geboten* | *gebeugt* |
|  | Literal | Zur Prostitution hat er das Mädchen… | *gezwungen* | *genötigt* | *geladen* |
| **Two-Argument Sentences – Passive Voice** | | | | | |
| 1 | Idiomatic | Von ihm wurde in den sauren Apfel … | *gebissen* | *gespuckt* | *geschnitten* |
|  | Literal | Von dem Hund wurde der Postbote bereits… | *gebissen* | *gezwickt* | *gerochen* |
| 2 | Idiomatic | Von ihnen beiden wurde ins gleiche Horn … | *geblasen* | *gehaucht* | *geseufzt* |
|  | Literal | Von dem talentierten Musiker wurde das Saxophon … | *geblasen* | *gepustet* | *geputzt* |
| 3 | Idiomatic | Von den Jungs wurde gemeinsam die Schulbank … | *gedrückt* | *gepresst* | *genutzt* |
|  | Literal | Von ihr wurden alle ihre Pickel wund… | *gedrückt* | *gekratzt* | *gelegen* |
| 4 | Idiomatic | Von ihr wurde immer nach den Sternen … | *gegriffen* | *gelangt* | *gelebt* |
|  | Literal | Von dem Mädchen wurde nach den Bonbons … | *gegriffen* | *gelangt* | *getastet* |
| 5 | Idiomatic | Von dem Jungen wurde schnell der Schnabel … | *gehalten* | *gestützt* | *geschüttelt* |
|  | Literal | Von mir wurde die Hundeleine sehr kurz … | *gehalten* | *gefasst* | *gebraucht* |
| 6 | Idiomatic | Von ihm wurde lange an der Strippe … | *gehangen* | *gependelt* | *gesprochen* |
|  | Literal | Von dem Regenschirm wurde lange am Haken … | *gehangen* | *gependelt* | *gewackelt* |
| 7 | Idiomatic | Von ihm wurde ordentlich auf den Putz … | *gehauen* | *gehackt* | *gestrichen* |
|  | Literal | Von ihm wurde kräftig auf den Tisch … | *gehauen* | *getrommelt* | *gemurmelt* |
| 8 | Idiomatic | Von dem jungen Mädchen wurde nach Blut … | *gelechzt* | *gegiert* | *geschielt* |
|  | Literal | Von dem durstigen Marathonläufer wurde nach Wasser … | *gelechzt* | *gegiert* | *gewühlt* |
| 9 | Idiomatic | Von der Frau wurde endlich der Schleier … | *gelüftet* | *gerafft* | *gestreift* |
|  | Literal | Von ihr wurde nie das miefende Zimmer … | *gelüftet* | *getrocknet* | *gemietet* |
| 10 | Idiomatic | Von ihr wurde der Mund zu voll… | *genommen* | *getan* | *gelassen* |
|  | Literal | Von der abhängigen Frau wurden die Tabletten … | *genommen* | *gefordert* | *gelassen* |
| 11 | Idiomatic | Von ihr wurde nach Tagen das Feld … | *geräumt* | *geleert* | *gekehrt* |
|  | Literal | Von dem Mädchen wurde letztendlich das Zimmer … | *geräumt* | *gewechselt* | *gemeint* |
| 12 | Idiomatic | Vom Teufel wurde mächtig das kleine Kind … | *geritten* | *gejagt* | *geärgert* |
|  | Literal | Von ihr wurde täglich das junge Pferd … | *geritten* | *gezügelt* | *gemocht* |
| 13 | Idiomatic | Von der jungen Frau wurde mächtig Schaum … | *geschlagen* | *gerpügelt* | *gebildet* |
|  | Literal | Von ihr wurde er aus purer Notwehr… | *geschlagen* | *geohrfeigt* | *geschützt* |
| 14 | Idiomatic | Von ihm wurde ganz alleine der Laden … | *geschmissen* | *geschwungen* | *gezahlt* |
|  | Literal | Von ihr wurde Wäsche durch das Haus… | *geschmissen* | *geschwungen* | *gefahren* |
| 15 | Idiomatic | Von ihm wurde nach Stunden frische Luft … | *geschnappt* | *gefangen* | *geatmet* |
|  | Literal | Von dem Frosch wurde gierig nach Fliegen … | *geschnappt* | *gehascht* | *geschaut* |
| 16 | Idiomatic | Von ihr wurden nach dem Unfall Sterne … | *gesehen* | *gesichtet* | *gefunden* |
|  | Literal | Von mir wurde ihr Bruder nie zuvor… | *gesehen* | *gefunden* | *gerettet* |
| 17 | Idiomatic | Von ihr wurde immer aufs falsche Pferd … | *gesetzt* | *gelehnt* | *gewollt* |
|  | Literal | Von ihnen wurden die neuen Pflanzenkeime bereits … | *gesetzt* | *gesät* | *gewollt* |
| 18 | Idiomatic | Von ihm wurde immer die zweite Geige … | *gespielt* | *gestimmt* | *gehört* |
|  | Literal | Von ihr wurde lange in einer Popband … | *gespielt* | *gewirkt* | *gedient* |
| 19 | Idiomatic | Von dem ehrgeizigen Redner wurde der Rahmen … | *gesprengt* | *geknackt* | *gestaltet* |
|  | Literal | Von den Bauarbeitern wurden die maroden Häuser … | *gesprengt* | *gestürzt* | *gemeldet* |
| 20 | Idiomatic | Von ihr wurde mutig ins kalte Wasser … | *gesprungen* | *gegangen* | *gerutscht* |
|  | Literal | Von dem Passanten wurde schnell zur Seite … | *gesprungen* | *gegangen* | *geflohen* |
| 21 | Idiomatic | Von dem kleinen Jungen wurde heimlich Schmiere … | *gestanden* | *gehockt* | *getrunken* |
|  | Literal | Von mir wurde lange in der Schlange … | *gestanden* | *gewartet* | *gelernt* |
| 22 | Idiomatic | Von ihm wurde jahrelang nach ihrer Pfeife … | *getanzt* | *gesteppt* | *gesucht* |
|  | Literal | Von ihnen wurde stundenlang zu klassischem Swing … | *getanzt* | *gesungen* | *geredet* |
| 23 | Idiomatic | Von der Psychologin wurde genau ins Schwarze … | *getroffen* | *gezielt* | *gestarrt* |
|  | Literal | Von ihm wurde Paul beim Kickboxen stark… | *getroffen* | *gefährdet* | *gefürchtet* |
| 24 | Idiomatic | Von dem Mann wurde schnell die Notbremse … | *gezogen* | *gelöst* | *gesichert* |
|  | Literal | Von ihnen wurde das Stahlseil noch fester … | *gezogen* | *gestrafft* | *gesichert* |
| **Three-Argument Sentences – Passive Voice** | | | | | |
| 25 | Idiomatic | Zur Kasse wurde ihr Freund von ihr... | gebeten | gedrängt | geholt |
|  | Literal | Um Hilfe wurde ihre Schwester von ihr... | gebeten | gefragt | gerufen |
| 26 | Idiomatic | Unter die Haube wurde sie von ihm… | gebracht | geführt | geschafft |
|  | Literal | Zur Post wurde der Brief von ihr… | gebracht | geschafft | gepackt |
| 27 | Idiomatic | Das Genick wurde ihm kräftig von ihr… | gebrochen | geteilt | geküsst |
|  | Literal | Der Arm wurde ihm vom Aufprall… | gebrochen | gestaucht | gewirbelt |
| 28 | Idiomatic | Aus der Hand wurde ihm von ihr… | gefressen | geschlungen | gewischt |
|  | Literal | Zum Frühstück wurden Mäuse von der Katze… | gefressen | gegessen | gezählt |
| 29 | Idiomatic | Auf den Zahn wurde ihm von ihr… | gefühlt | gespürt | gescheint |
|  | Literal | Den Puls wurde dem Patienten von ihr… | gefühlt | gespürt | geregelt |
| 30 | Idiomatic | Ins Feuer wurde mächtig Öl von ihm … | gegossen | gesondert | gefüllt |
|  | Literal | Die Blumen wurden der Nachbarin von ihr… | gegossen | gewässert | gepflegt |
| 31 | Idiomatic | In die Karten wurde ihm von ihr… | geguckt | gespäht | gemalt |
|  | Literal | Nach Autos wurde für ihn von ihr… | geguckt | gespäht | gehorcht |
| 32 | Idiomatic | An den Nagel wurde es von ihm… | gehängt | gebaumelt | gesteckt |
|  | Literal | Auf Kleiderbügel wurden die Hemden von mir… | gehängt | gebaumelt | gesteckt |
| 33 | Idiomatic | In den Himmel wurde sie von ihm… | gehoben | gewuchtet | geflogen |
|  | Literal | Der schwere Karton wurde ihr von ihm… | gehoben | geschleppt | geschaukelt |
| 34 | Idiomatic | Im Sack wurde die Katze von ihm… | gekauft | gehandelt | getragen |
|  | Literal | Neue Klamotten wurden ihm wieder von ihr… | gekauft | gehandelt | gestrickt |
| 35 | Idiomatic | Auf die Finger wurde ihm von ihr… | geklopft | gehämmert | geleuchtet |
|  | Literal | An die Tür wurde ihr von ihm… | geklopft | gepocht | gepinselt |
| 36 | Idiomatic | Ein Haar wurde ihr nie von ihm… | gekrümmt | gebogen | gerupft |
|  | Literal | Die Banane wurde uns von der Natur… | gekrümmt | gebogen | gezeugt |
| 37 | Idiomatic | Aufs Eis wurde die Angestellte von ihm… | gelegt | gestellt | gewählt |
|  | Literal | Um sie wurde der Arm von ihm… | gelegt | gemacht | geworfen |
| 38 | Idiomatic | Die Augen wurden der Freundin von ihm… | geöffnet | geklärt | gebessert |
|  | Literal | Die Schmuckschatulle wurde ihr vorsichtig von ihm… | geöffnet | geklappt | geschätzt |
| 39 | Idiomatic | Das Wasser wurde ihr nicht von ihm… | gereicht | gegeben | geprüft |
|  | Literal | Der Brotkorb wurde dem Mann von ihr… | gereicht | gegeben | gestattet |
| 40 | Idiomatic | Aus der Bahn wurde sie von ihm… | geschleudert | geschmettert | gelenkt |
|  | Literal | In die Luft wurde Konfetti von ihr… | geschleudert | geschmettert | gestapelt |
| 41 | Idiomatic | Ins Herz wurde er sehr von ihr… | geschlossen | gesperrt | gezeichnet |
|  | Literal | Das Fenster wurde der Frau von ihm… | geschlossen | gekippt | gebaut |
| 42 | Idiomatic | Aufs Abstellgleis wurde die Frau von ihm… | geschoben | gerückt | gebunden |
|  | Literal | Der Wagen wurde der Oma von ihm… | geschoben | gerollt | geordnet |
| 43 | Idiomatic | Auf die Folter wurde sie von ihm… | gespannt | gedehnt | gefesselt |
|  | Literal | An den Wagen wurden die Pferde von ihm… | gespannt | gekettet | gewöhnt |
| 44 | Idiomatic | Aufs Dach wurde der Geliebten von ihm… | gestiegen | geklettert | gefolgt |
|  | Literal | Auf den Rücken wurde Pferden von ihm… | gestiegen | gekrabbelt | gefallen |
| 45 | Idiomatic | Vor den Kopf wurde sie von ihm… | gestoßen | gerammt | gesenkt |
|  | Literal | Ins Herz wurde der Dolch von ihm… | gestoßen | gebohrt | gefügt |
| 46 | Idiomatic | Die Flügel wurden dem Jungen von ihr… | gestutzt | gekürzt | gekämmt |
|  | Literal | Den Bart wurde ihm morgens von ihr… | gestutzt | getrimmt | gefärbt |
| 47 | Idiomatic | Auf die Zehen wurde ihr von ihm… | getreten | getrampelt | gerast |
|  | Literal | In die Hacken wurde ihm von ihr… | getreten | gestampft | gehüpft |
| 48 | Idiomatic | In die Enge wurde sie von ihm… | getrieben | gehetzt | gekämpft |
|  | Literal | Heimwärts wurden die Schafe vom Hund… | getrieben | geleitet | gelockt |
| 49 | Idiomatic | Den Kopf wurde ihm gründlich von ihr… | gewaschen | gereinigt | gemessen |
|  | Literal | Die Haare wurden der Tochter von ihm… | gewaschen | gesäubert | gebürstet |
| 50 | Idiomatic | In die Schranken wurde er von ihr… | gewiesen | geschickt | geneigt |
|  | Literal | Die Tür wurde ihr wütend von ihm… | gewiesen | geschildert | geliefert |
| 51 | Idiomatic | Die Krallen wurden ihm von der Frau… | gezeigt | gedeutet | genehmigt |
|  | Literal | Den Weg wurde dem Touristen von ihr… | gezeigt | gedeutet | geflutet |
| 52 | Idiomatic | In die Knie wurde er von ihr… | gezwungen | geboten | gebeugt |
|  | Literal | Zur Prostitution wurde das Mädchen von ihm… | gezwungen | genötigt | geladen |

**Appendix D**

*Fixed Effects of the Predictors in the Linear Mixed-Effect Model for Log-Transformed Reaction Times in Experiment 2.*

|  | **Estimate** | **Std. Error** | **df** | ***t-*value** | ***p*** |
| --- | --- | --- | --- | --- | --- |
| Intercept: literal, active, three, 6 letters | 6.882 | 0.15 | 108 | 44.67 | < .0001 |
| Sentence (idiomatic) | -0.116 | 0.06 | 115 | -1.97 | .0516 |
| Voice (passive) | 0.054 | 0.02 | 2828 | 2.36 | .0182 |
| Arguments (two) | -0.040 | 0.06 | 115 | -0.65 | .5149 |
| Target Length (per additional letter) | 0.046 | 0.02 | 99 | 2.59 | .0111 |
| Sentence (i) × Voice (p) | 0.034 | 0.03 | 2828 | 1.07 | .2871 |
| Sentence (i) × Arguments (two) | 0.030 | 0.09 | 115 | 0.35 | .7257 |
| Voice (p) × Arguments (two) | -0.001 | 0.03 | 2828 | -0.02 | .9811 |
| Sentence (i) × Voice (p) × Arguments (two) | -0.126 | 0.05 | 2828 | -2.67 | .0077 |

*Note.* The intercept refers to literal sentences in active voice with three arguments and targets with 6 letters.

**Appendix E**

*Fixed Effects of the Predictors in the Linear Mixed-Effect Model for Log-Transformed Reaction Times in the Post-Hoc Analysis.*

|  | | **Estimate** | **Std. Error** | **df** | ***t*-value** | ***p*** | **AIC** |
| --- | --- | --- | --- | --- | --- | --- | --- |
| *Analysis with Adjacency* | |  |  |  |  |  | 4416 |
|  | Intercept | 6.810 | 0.10 | 231 | 65.15 | < .0001 |  |
|  | Sentence Type (idiomatic) | -0.147 | 0.03 | 271 | -4.81 | < .0001 |  |
|  | Voice (passive) | 0.077 | 0.01 | 5777 | 9.15 | < .0001 |  |
|  | **Adjacency (nonadjacent)** | 0.053 | 0.02 | 5778 | 3.14 | .0017 |  |
|  | Target Length (per additional letter) | 0.042 | 0.01 | 203 | 3.45 | .0007 |  |
|  | Voice (passive) × Arguments (two) | 0.077 | 0.02 | 5370 | 2.98 | .0029 |  |
| *Analysis with Arguments* | |  |  |  |  |  | 4468 |
|  | Intercept | 6.836 | 0.11 | 227 | 64.71 | < .0001 |  |
|  | Sentence Type (idiomatic) | -0.112 | 0.03 | 202 | -3.93 | .0001 |  |
|  | Voice (passive) | 0.078 | 0.01 | 5779 | 9.20 | < .0001 |  |
|  | **Arguments (two)** | -0.001 | 0.03 | 202 | -0.02 | .9859 |  |
|  | Target Length (per additional letter) | 0.041 | 0.01 | 202 | 3.41 | .0008 |  |

*Note.* The intercept in the analysis with adjacency refers to literal sentences in active voice with adjacent arguments and targets with 6 letters; the intercept in the analysis with arguments refers to literal sentences in active voice with three arguments and targets with 6 letters.
